# Comparative morphology of the nervous system in three phylactolaemate bryozoans

**DOI:** 10.1186/s12983-015-0112-2

**Published:** 2015-10-12

**Authors:** Ksenia V. Shunkina, Olga V. Zaytseva, Viktor V. Starunov, Andrew N. Ostrovsky

**Affiliations:** Laboratory of Evolutionary Morphology, Zoological Institute, Russian Academy of Sciences, Universitetskaja nab. 1, 199034 Saint Petersburg, Russia; Department of Invertebrate Zoology, Faculty of Biology, Saint Petersburg State University, Universitetskaja nab. 7/9, 199034 Saint Petersburg, Russia; Department of Palaeontology, Faculty of Earth Sciences, Geography and Astronomy, Geozentrum, University of Vienna, Althanstraße 14, A-1090 Vienna, Austria

**Keywords:** Nervous system, Immunohistochemistry, Neuromediators, Phylactolaemata, Bryozoa

## Abstract

**Background:**

Though some elements of the bryozoan nervous system were discovered 180 years ago, few studies of their neuromorphology have been undertaken since that time. As a result the general picture of the bryozoan nervous system structure is incomplete in respect of details and fragmentary in respect of taxonomic coverage.

**Results:**

The nervous system of three common European freshwater bryozoans – *Cristatella mucedo*, *Plumatella repens* (both with a horseshoe-shaped lophophore) and *Fredericella sultana* (with a circular lophophore) had numerous differences in the details of the structure but the general neuroarchitecture is similar. The nervous system of the zooid consists of the cerebral ganglion, a circumpharyngeal ring and lophophoral nerve tracts (horns), both sending numerous nerves to the tentacles, and the nerve plexuses of the body wall and of the gut. A number of the important details (distal branching of the additional radial nerve, pattern of distribution of nerve cells and neurites in the ganglion, etc.) were described for the first time. The number and position of the tentacle nerves in *Cristatella mucedo* was ascertained and suggestions about their function were made. The revealed distribution of various neuromediators in the nervous system allowed us to suggest functional affinities of some major nerves.

**Conclusions:**

Despite the basic similarity, both the ganglion and the lophophore nervous system in Phylactolaemata have a more complex structure than in marine bryozoans (classes Gymnolaemata and Stenolaemata). First of all, their neuronal network has a denser and more complex branching pattern: most phylactolaemates have two large nerve tracts associated with lophophore arms, they have more nerves in the tentacles, additional and basal branches emitting from the main radial nerves, etc. This, in part, can be explained by the horseshoe shape of the lophophore and a larger size of the polypide in freshwater species. The structure of the nervous system in *Fredericella sultana* suggests that it underwent a secondary simplification following the reduction of the lophophore arms. Colony locomotion in *Cristatella mucedo* is based on co-ordinated activity of two perpendicular muscle layers of the sole and the plexus of motor neurons sandwiched between them. The trigger of this activity and the co-ordination mechanism remain enigmatic.

**Electronic supplementary material:**

The online version of this article (doi:10.1186/s12983-015-0112-2) contains supplementary material, which is available to authorized users.

## Introduction

Bryozoans are sessile filter-feeding invertebrates whose sheet-like, bushy or arborescent colonies are abundant in various freshwater and marine bottom habitats, from rivers and lakes to a shallow subtidal and oceanic abyss. Providing shelter and food for numerous hydrobionts, bryozoans, together with sponges and cnidarians, are considered among the most important elements in the various benthic communities [1].

Each bryozoan colony consists of interconnected modules, zooids, which are formally subdivided into the polypide – a protruding ciliated tentacle crown (lophophore) with a digestive tract and associated musculature and the cystid – the receptacle of the polypide. The external layer (ectocyst) of the cystid wall of marine bryozoans (classes Gymnolaemata and Stenolaemata) can be calcified or chitinous, whereas in the freshwater class Phylactolaemata it is chitinous or gelatinous. The ectocyst overlays the endocyst consisting of epidermis and the circular and longitudinal muscle layers underlain by peritoneum in phylactolaemates. In gymno- and stenolaemates the muscle layers and, sometimes, peritoneal lining are missing. Both gymnolaemates and stenolaemates possess a bell-shaped tentacle crown with a central mouth, the tentacle bases forming a circle around it. In contrast, in most phylactolaemates the tentacles are arranged along the lophophore arms and around the eccentric mouth in a horseshoe pattern [2, 3].

**Fig. 1:**
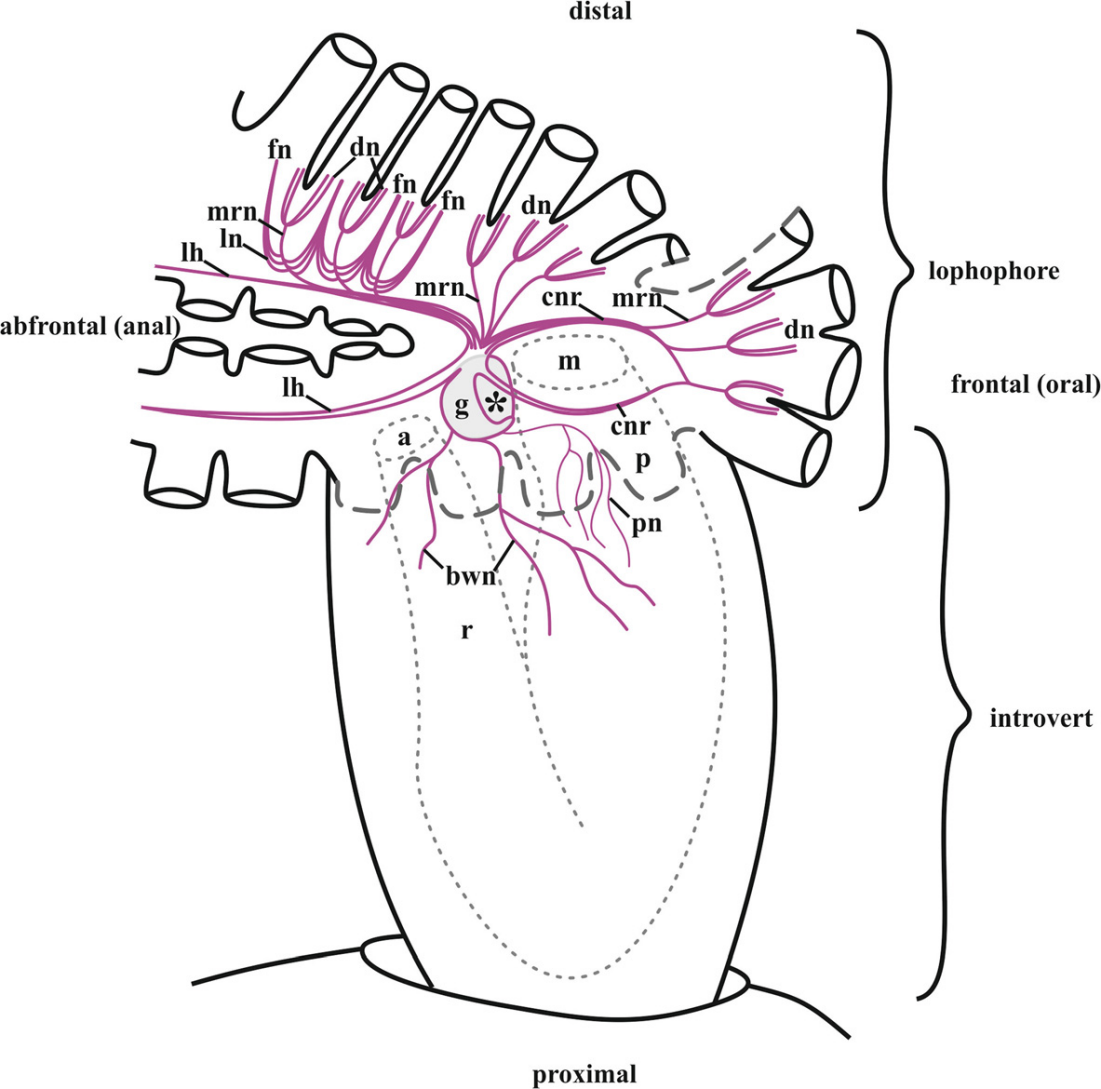
Simplified scheme of zooidal innervation in *Cristatella mucedo* based on staining with antibodies against acetylated tubulin (only some tentacle bases shown, partially by dashed line). Cavity of the cerebral ganglion shown by asterisk. Abbreviations: a, anus; bwn, nerves of the body wall originating from the basal nerves of cerebral ganglion (partially shown, paired branches on the opposite side are not shown); cnr, circumpharyngeal nerve ring; dn, distal branches of main radial nerve; fn, tentacle frontal nerve; g, cerebral ganglion; lh, lophophore nerve tracts (horns); ln, lateral branches of main radial nerve; m, mouth; mrn, main radial nerve; p, pharynx; pn, nerves of pharynx (paired branch on the opposite side is not shown); r, rectum

The term “lophophore” (from Greek “tuft- or crest-[of tentacles]-bearer”), coined by Allman [4], is commonly used in the bryozoological literature as a general term for the tentacle crown. However, while some authors apply it to the tentacle-bearing area around the mouth (as Allman originally did), others use it to describe the entire tentacle crown, either without the eversible part of the body wall (introvert) that supports the tentacles, or together with it [3–6]. We followed the second variant, applying “lophophore” to the tentacle crown without the introvert (Fig. 1). Introvert is everted and inverted together with the excursions of the tentacle crown. When the lophophore is retracted, the introvert’s wall surrounds the tentacles and is referred to as a tentacle sheath. Its wall continues to that of the vestibulum – a small chamber between the zooidal opening and the tentacle sheath. The vestibulum is a part of the cystid wall since it does not degenerate. The tentacle sheath belongs to the polypide since it degenerates together with the tentacle crown and the gut. Both the introvert and the vestibulum are parts of the zooidal body wall [3].

**Fig. 2:**
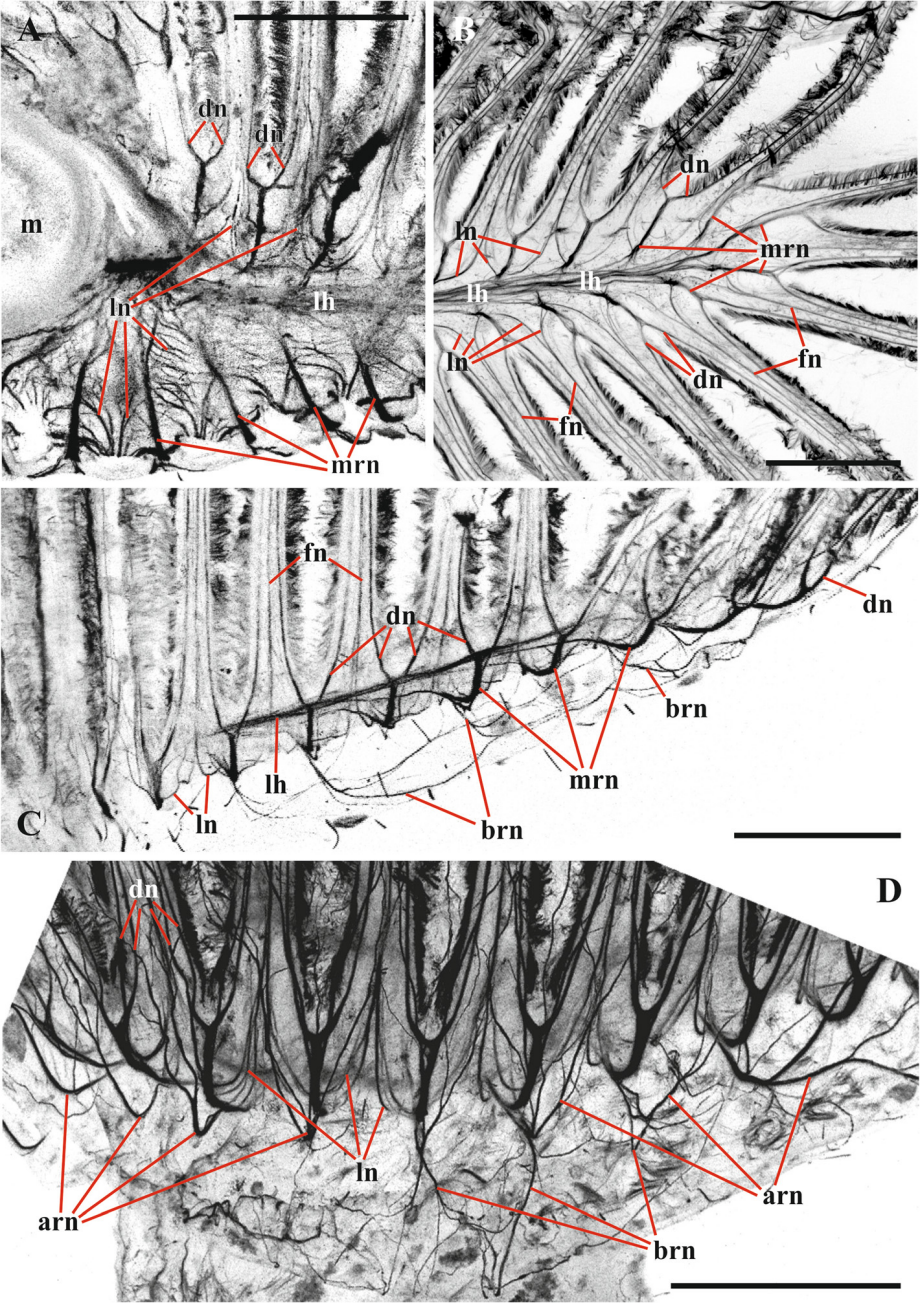
Lophophore innervation in *Cristatella mucedo* visualized after staining with antibodies against acetylated tubulin (**a**, **b** arm view from above, double distal dichotomy of the main radial nerves not seen in this plane; **c**, **d** lateral view, double dichotomy of the main radial nerves well seen in **d**). **a** Proximal part of the arm with basal parts of the tentacles (mouth region seen on the left); **b** distal part of the arm; **c** distal part of the arm with basal parts of the tentacles (lophophore horn is behind the main radial nerves); **d** fronto-lateral part of the lophophore showing basal parts of tentacles. Abbreviations: arn, additional radial nerve; brn, basal radial nerve; dn, distal branches of main radial nerve; fn, tentacle frontal nerve; lh, lophophore nerve tract (horn); ln, lateral branches of main radial nerve; m, mouth; mrn, main radial nerve. Scale bars: 100 μm

**Fig. 3:**
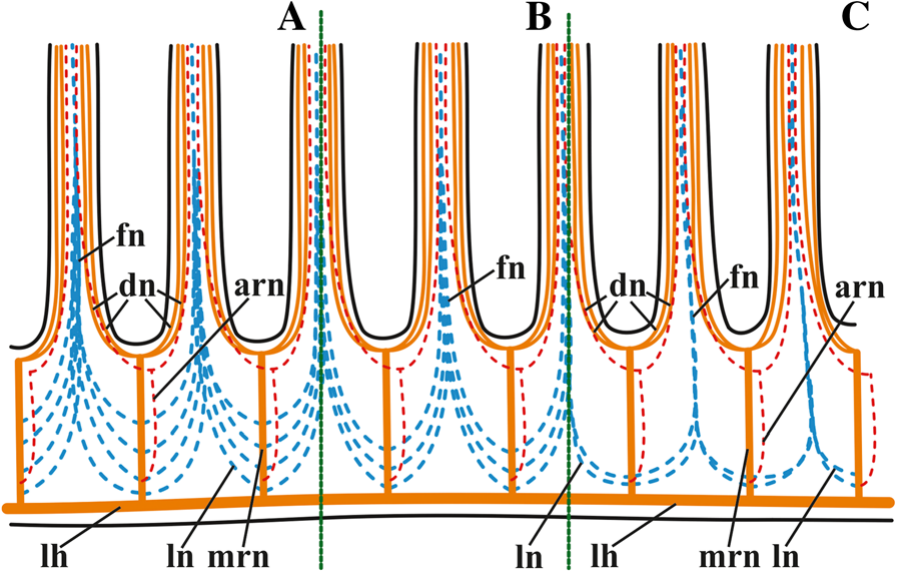
Simplified scheme of lophophore base innervation in *Cristatella mucedo* (**a**) *Plumatella repens* (**b**) and *Fredericella sultana* (**c**) based on staining with antibodies against acetylated tubulin. Abbreviations: arn, additional radial nerve; dn, distal branches of main radial nerves; fn, frontal nerve; lh, lophophore nerve tracts (horns); ln, lateral branches of main radial nerves; mrn, main radial nerves

**Fig. 4:**
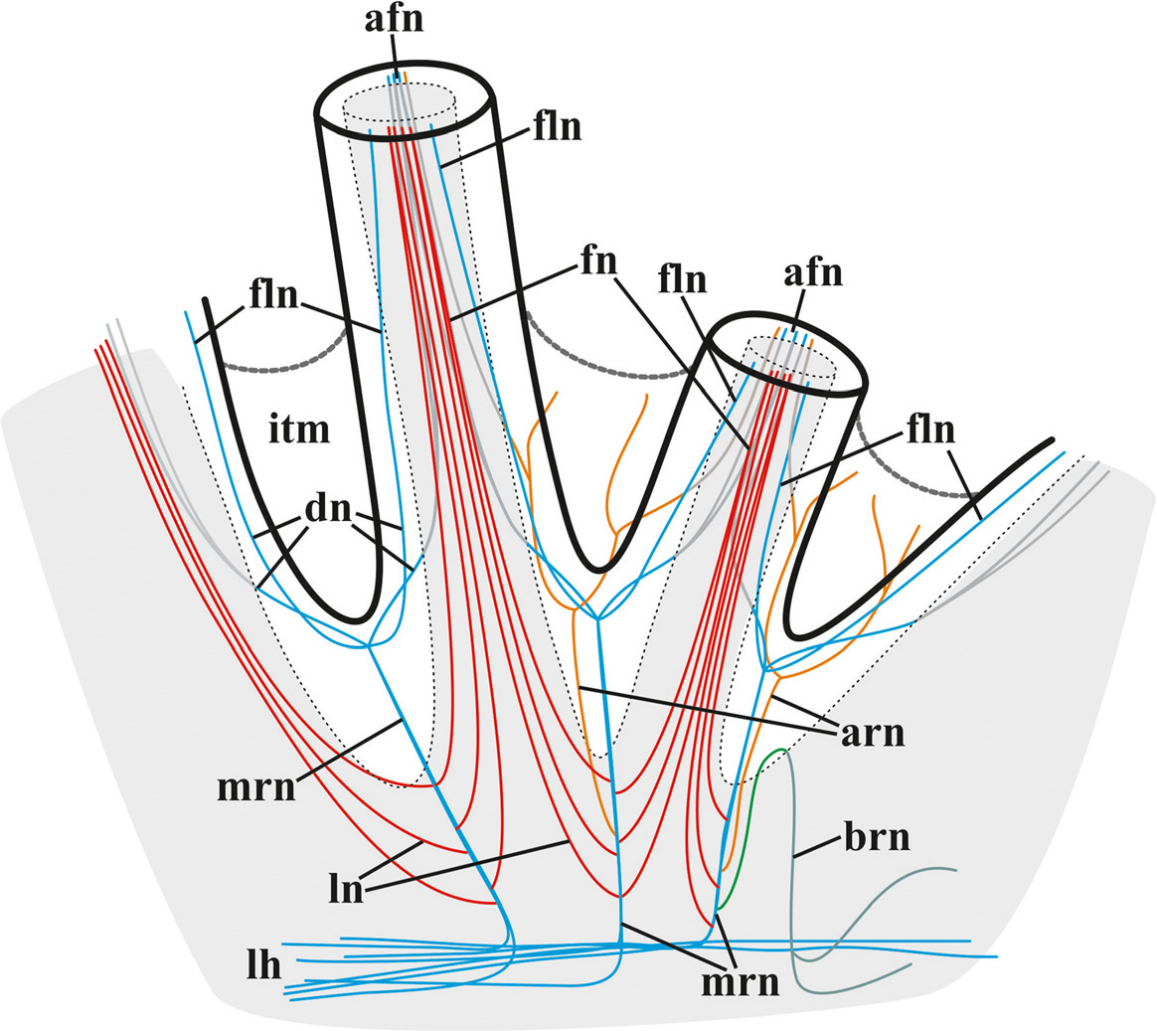
Schematic 3D-reconstruction of lophophore and tentacle innervation in *Cristatella mucedo* based on staining with antibodies against acetylated tubulin and TEM. Abbreviations: afn, tentacle abfrontal nerve; arn, additional radial nerve; brn, basal radial nerve; dn, distal branches of main radial nerve; fln, tentacle frontolateral nerve; fn, tentacle frontal nerve; itm, intertentacular membrane; lh, lophophore nerve tract (horn); ln, lateral branches of main radial nerves; mrn, main radial nerve (abfrontolateral nerves not shown)

Despite their miniature size, anatomical simplicity and a mostly immobile lifestyle Bryozoa demonstrate an outstanding diversity of behavioural reactions. The lophophores may perform various individual and group activities associated with feeding, colony cleaning and spawning [7–16]. A few highly unusual mobile colonies, which may creep or “walk” along the bottom, have evolved several times independently in this group [17].

If we are to understand both these phenomena – a complicated lophophore behaviour and colony locomotion – we need detailed knowledge of both zooidal and colonial neuromorphology. In addition, the comparative studies on the structure of the nervous system in the lophophores of different shape could provide an insight in their evolution. Studies of the bryozoan nervous system date back to the first half of 19^th^ century but, as it often happens, only a handful of species has been described in this respect. Thus, information on bryozoan neuromorphology is fragmentary and does not add up to a coherent overall picture. Moreover, even the available data require careful checking since they were obtained with the use of different staining methods, and were often based on 2D-preparations, which makes comparisons difficult.

Initially, bryozoan internal structure was studied using total preparations (alive or fixed) without staining. Works of Dumortier and Van Beneden [18–22], Allman [23, 24], Hancock [25], Nitsche [26] and Hyatt [27] provided the first descriptions of the phylactolaemate nervous system, obtained during the studies of the common freshwater bryozoans from the genera *Lophopus*, *Fredericella, Plumatella* and *Cristatella* (see Additional file 1 for the History of the research). Supraoesophagal position of the cerebral ganglion has been revealed together with the presence of the circumpharyngeal nervous ring and two lophophoral nerve tracts (later on called ‘horns’ in the species with the horse-shoe lophophore) issuing numerous branches towards the spaces between the tentacles. Allman [23] discovered a cavity inside a ganglion, and Hyatt [27] described the paired nervous branches going from the ganglion towards the lophophoral arms, epistome, tentacle sheath and the gut.

Anatomical studies greatly benefited from the invention of the histological methods. Using them, several authors described the nervous system in a number of phylactolaemates [28–33]. Following Hyatt [27], Braem [34, 35] described and depicted the general neuroanatomy in the species with horseshoe and circular lophophores, showing that *Fredericella*, though having circular tentacle crown, has the rudimentary lophophore tracts. At that time most authors considered Phylactolaemata as a basal (and stem) bryozoan group originated from phoronids possessing a horseshoe-shaped lophophore and a tripartite coelom. In contrast, a few other researchers believed that this clade is a descendant of the marine bryozoans (reviewed in [5, 36, 37]). The circular lophophore of *Fredericella* was used as an important argument in favour of the second hypothesis [29]. Rudimentary lophophore tracts found by Braem might indicate that it is just a modified phylactolaemate (see also below).

Such a discussion would be impossible without studies of the marine Bryozoa that began when Van Beneden [38, 39] described the cerebral ganglion in two gymnolaemates. While no special works on gymnolaemate neuroanatomy were published during the second half of the 19^th^ century, some information on it can be found in several taxonomical and anatomical papers [29, 40–45] (see also Additional file 1).

An important milestone were two papers of Gerwerzhagen [46, 47] who studied the nervous system in both phylacto- and gymnolaemates (one species of each group) using histological sections and vital staining by methylen blue. In addition to a very detailed descriptive work, this author widely discussed the functional affinities of various nerves based on their position. This functional approach was further developed by several authors [48–54] including Lutaud [55–66] who, in addition to various staining methods, applied transmission electron microscopy (TEM) to the studies of the bryozoan nervous system (reviewed in [66, 67]). The next step was an application of immunohistochemical methods. During the last decade a few studies on the nerve elements of the larval [68–72], ancestrular [73] and adult stages [74–78] of Bryozoa have been published, but despite this progress, the general picture is still incomplete.

The reason for this unsatisfactory state is that detailed studies are rare, and the nervous system of most species has been described rather fragmentarily. Phylactolaemates *Cristatella mucedo* and *Lophopus crystallinus*, and gymnolaemate *Electra pilosa* [46, 52, 56, 57] are exceptions but they were studied before the invention of confocal microscopy. Although some recent studies present careful description of the neuroanatomy of phylactolaemate, *Fredericella sultana* [74] and gymnolaemates, *Hislopia malayensis* and *Paludicella articulata* [75, 78], the lack of a more complete taxonomic coverage is the largest obstacle to getting a broad comparative picture of bryozoan nervous system and its functions.

What is important (and also intriguing) in this respect is that different bryozoan species – although from the same classes and orders – were described as having remarkable differences in neuromorphology. These differences become obvious, for instance, when comparing descriptions of Gerwerzhganen [46] and Marcus [52] of the above mentioned *C. mucedo* and *L. crystallinus*. One gets a similar impression after comparison of published descriptions and schemes on gymnolaemates [47, 51, 53]. The cause of these differences is currently unclear (they might be associated with interspecific variability, the use of different staining methods, or both), and since in all these cases the research was predominantly based on histological methods, it clearly should be checked and updated using modern techniques. Also, the use of antibodies to various neuromediators should help one to ascertain the functional meaning of the nerve elements. In an attempt to fill this gap at least partially, we used antibody staining to visualize the nervous system in three species of common European freshwater bryozoans, aiming:

1. to provide detailed comparative descriptions of their nervous system;
2. to suggest hypotheses about the functions of different nerve structures based on the distribution of the common neuromediators;
3. to compare the nervous system of Phylactolaemata with that of Gymnolaemata based on our own results and literature data.

## Results

For our comparative study we selected three phylactolaemate species: *Cristatella mucedo* (Cuvier, 1798), *Plumatella repens* (Linnaeus, 1758) and *Fredericella sultana* (Blumenbach, 1779). They are among the most common freshwater bryozoans in Europe. These species differ greatly in both colonial and zooidal gross morphology. *P. repens* and *F. sultana* have encrusting branching colonies permanently attached to the substrate and tubular cystids with a sclerotized chitinous ectocyst (plumatellid colony type). *C. mucedo* has oval (when young) or worm-shaped (when mature) colonies with a gelatinous ectocyst. These bryozoans are well-known for their unique ability to move along the substrate (lophopodid colony type). The basal wall of the colony is transformed into a locomotory organ, the sole. Whereas polypides of *C. mucedo* and *P. repens* have a horseshoe-shaped lophophore with 30–60 tentacles, *F. sultana* has a small bell-shaped lophophore similar to that in marine bryozoans (with 20–23 tentacles depending on the polypide age). The tentacle crown diameter in *Fredericella* (measured as the diameter of the circle formed by the tentacle tips in feeding position) was about 1 mm. In *Cristatella* and *Plumatella* the tentacle tips formed an oval with the dimensions of about 1 × 1.5 mm.

For the sake of convenience, the nervous system of three phylactolaemates under study is described as that of (1) the lophophore, (2) the tentacle sheath (introvert) and the vestibulum, (3) the cystid wall and (4) the digestive tract. To highlight the differences between three species, the nervous system of *Cristatella mucedo* is described first in each section followed by comparative descriptions of two other species. General neuromorphology was described using antibodies against acetylated α-tubulin. Antibodies to serotonin and FMRFamide were used to visualize the distribution of neuromediators. Some details in *C. mucedo* were verified by catecholamine staining and TEM.

### Lophophore

**Fig. 5:**
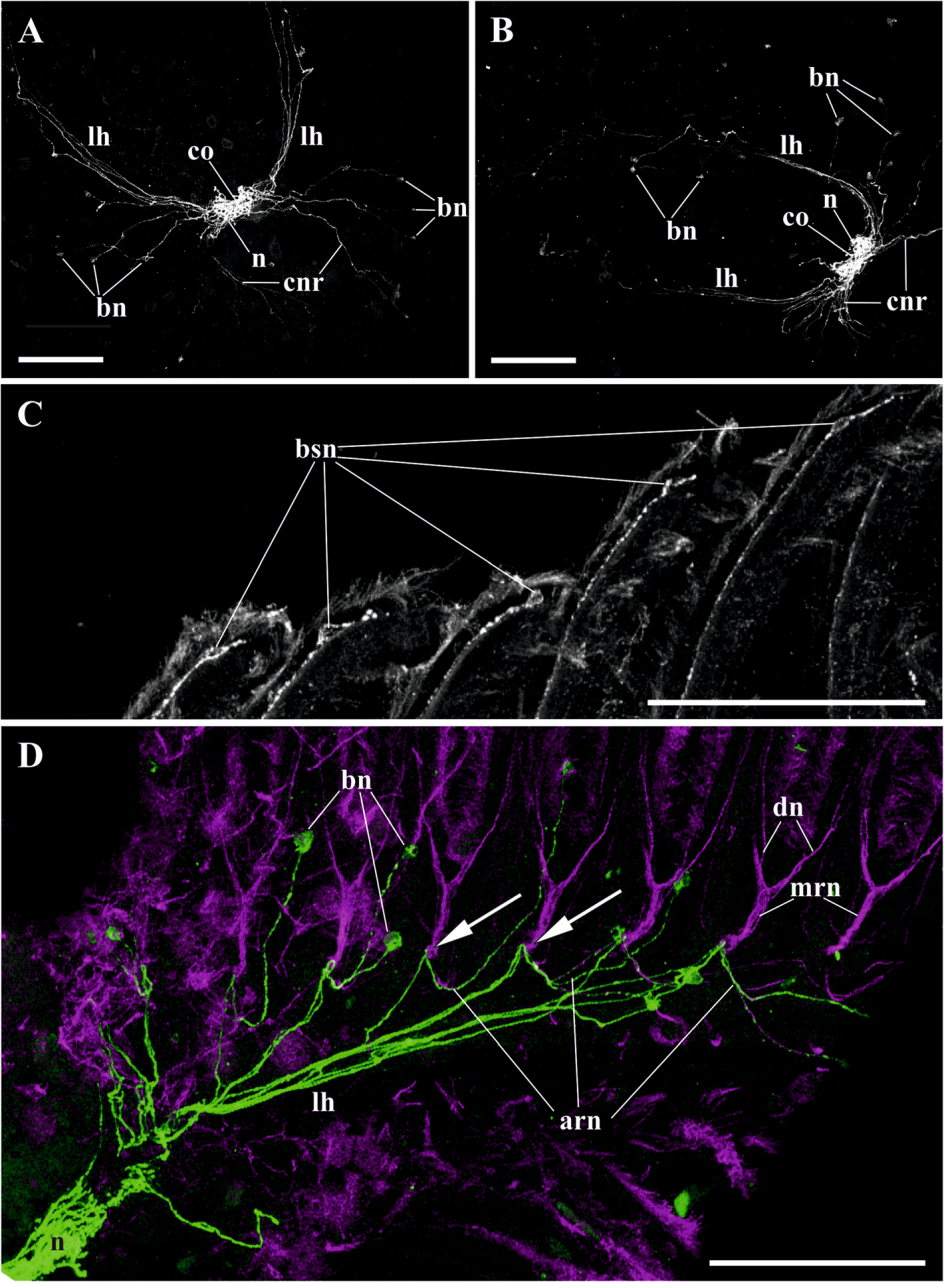
Details of lophophore innervation and cerebral ganglion in *Cristatella mucedo*. **a**, **b** cerebral ganglion and lophophoral nerves (view from above, bipolar neurons situated near oral and lophophore arms tentacle bases, in some of them the main neurites merge before joining main radial nerves in **a**) (staining with antibodies against serotonin); **c** tentacle tips with sensory bipolar neurons which central neurite descends with tentacle frontal nerve (staining with antibodies against FMRFamide); **d** innervation of lophophore arm (view from above, bipolar neurons situated near tentacle bases, arrows point to the places where additional radial nerves join main radial nerves) (staining with antibodies against serotonin shown in green and against α-tubulin in violet). Abbreviations: arn, additional radial nerve; bn, bipolar neurons; bsn, bipolar sensory neurons situated near the tentacle tip; cnr, serotonin-like immunoreactive neurites of circumpharyngeal nerve ring; co, commissure; dn, distal branches of main radial nerve; lh, lophophore nerve tract (horn); mrn, main radial nerve; n, neuropil of cerebral ganglion. Scale bars: 100 μm

**Fig. 6:**
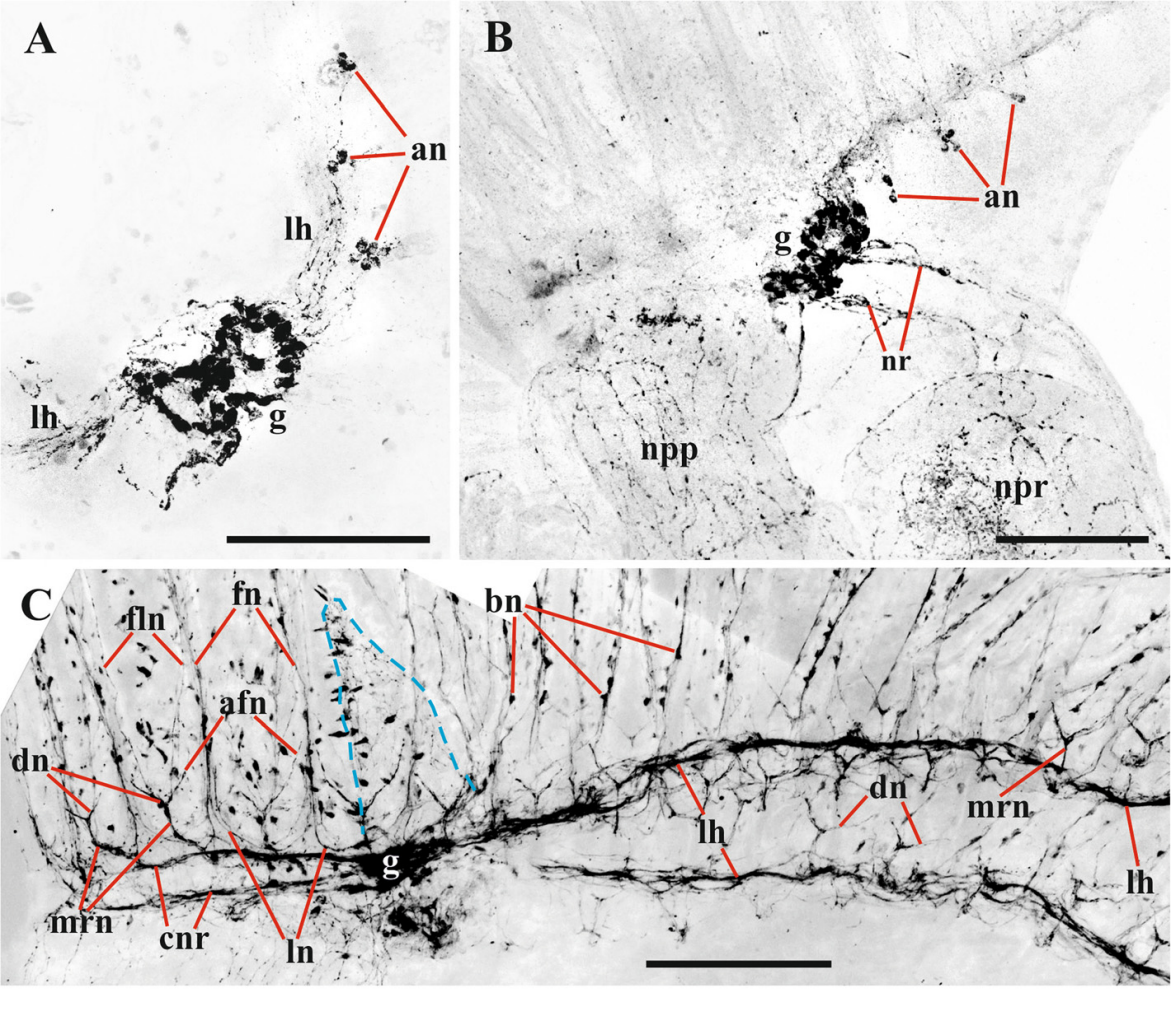
Details of lophophore innervation and cerebral ganglion in *Cristatella mucedo*. **a**, **b** cerebral ganglion (**a** view from above; **b** lateral view) (staining with antibodies against FMRFamide); **c** lophophore innervation (lateral view; epistome is outlined by blue colour) (catecholamine staining). Abbreviations: afn, tentacle abfrontal nerve; an, accumulations of neurons near bases of lateral tentacles; bn, bipolar neurons of tentacles; cnr, circumpharyngeal nerve ring; dn, distal branches of main radial nerve; fln, tentacle frontolateral nerve; fn, tentacle frontal nerve; g, cerebral ganglion; lh, lophophore nerve tract (horn); ln, lateral branches of main radial nerves; mrn, main radial nerve; npp, nerve plexus of pharynx; npr, nerve plexus of rectum; nr, nerve strands emitting from cerebral ganglion to rectum. Scale bars: 100 μm

A small (70.0–80.0 × 30.0–40.0 μm in diameter, *n* = 5) cerebral ganglion of *C. mucedo* is situated on the abfrontal (dorsal in old literatutre) wall of the pharynx (Figs. 1, 5a, b, d, 6, 14c, 15b, 16 a, b, *g*). It consists of a peripheral zone predominantly composed by the neuron cell bodies, a neuropil of both thin and thick nerve projections, and a small distal lumen inside the neuropil. Most neurons in the peripheral zone are situated in the ganglion basal part. The neuropil constitutes the anal (faced to anus) and the basal parts of the ganglion.

**Fig. 7:**
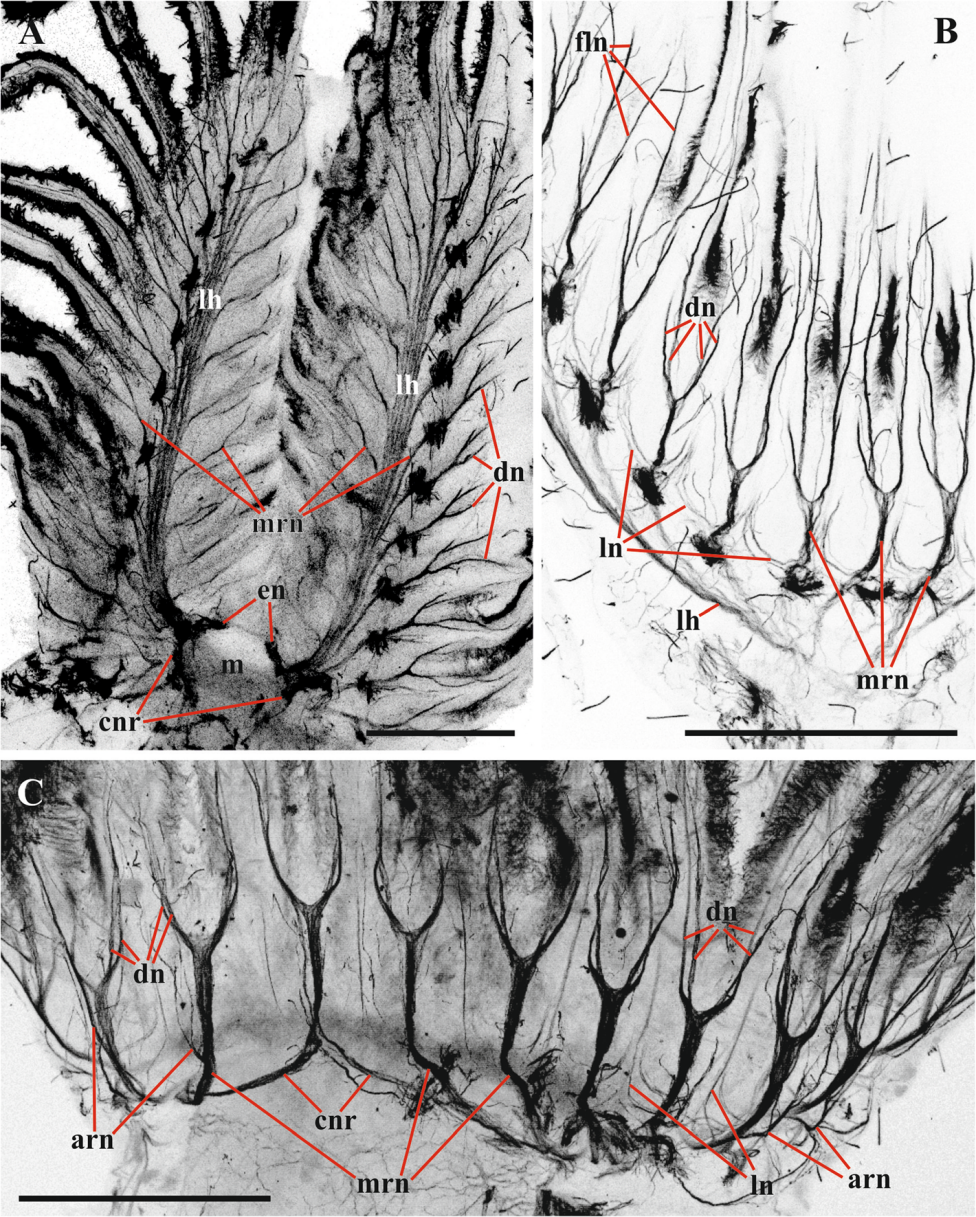
Lophophore innervation in *Plumatella repens* visualized after staining with antibodies against acetylated tubulin. **a**, lophophore view from above with the mouth area seen below; **b** lateral view of arm; **c** lophophore view from frontal side showing ‘closure’ of circumpharyngeal nerve ring (main radial nerves are emitted from circumpharyngeal nerve ring). Abbreviations: arn, additional radial nerve; cnr, circumpharyngeal nerve ring; dn, distal branches of main radial nerve; en, nerves of epistome; fln, tentacle frontolateral nerve; lh, lophophore nerve tract (horn); ln, lateral branches of main radial nerve; m, area of mouth; mrn, main radial nerve. Scale bars: 100 μm

**Fig. 8:**
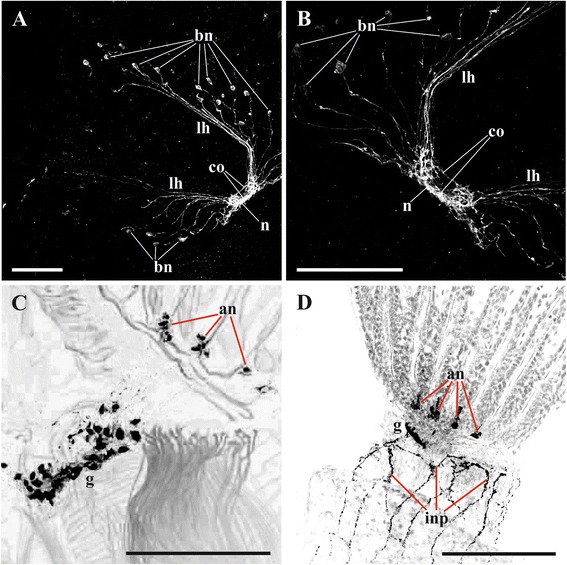
Details of lophophore innervation and cerebral ganglion in *Plumatella repens* and *Fredericella sultana*. **a**, **b**, cerebral ganglion and lophophoral nerves in *P. repens* (view from above) (staining with antibodies against serotonin); **c**, cerebral ganglion and accumulations of neurons at the tentacle bases in *P. repens* (lateral view) (staining with antibodies against FMRFamide); **d**, cerebral ganglion and accumulations of neurons at the tentacle bases in *F. sultana* (lateral view) (staining with antibodies against FMRFamide). Abbreviations: an, accumulations of neurons at tentacle bases; bn, bipolar neurons situated near oral and lophophore arms tentacle bases; co, commissure; g, cerebral ganglion; inp, nerve plexus of introvert; lh, lophophore nerve tract (horn); n, neuropil of cerebral ganglion. Scale bars: 100 μm

**Fig. 9:**
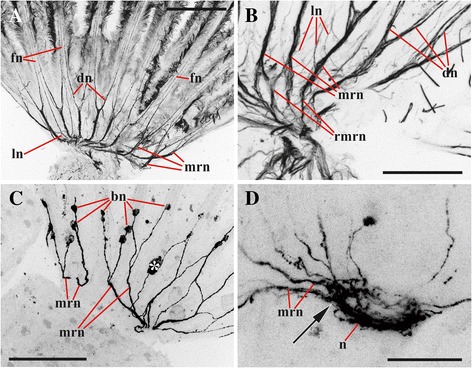
Details of lophophore innervation and cerebral ganglion in *Fredericella sultana*. **a**, **b** innervation of lophophore base and proximal parts of tentacles (lateral view) (in **b** roots of main radial nerves are clearly seen) (staining with antibodies against acetylated tubulin); **c** lophophore innervation (view from oral side showing that circumpharyngeal nerve ring is incomplete and that main neurites of the adjacent bipolar nerves merge together before joining the radial nerves) (staining with antibodies against serotonin); **d** cerebral ganglion (view from anal side, arrow shows the local area corresponding to lophophore horn from which main radial nerves are emitted) (staining with antibodies against serotonin). Abbreviations: bn, bipolar neurons situated inside tentacle bases; dn, distal branches of main radial nerve; fn, tentacle frontal nerve; ln, lateral branches of main radial nerve; mrn, main radial nerve; n, neuropil of cerebral ganglion; rmrn, roots of main radial nerves. *indicates a visual artifact. Scale bars: (**a**, **c**) 100 μm, (**b**, **d**) 50 μm

**Fig. 10:**
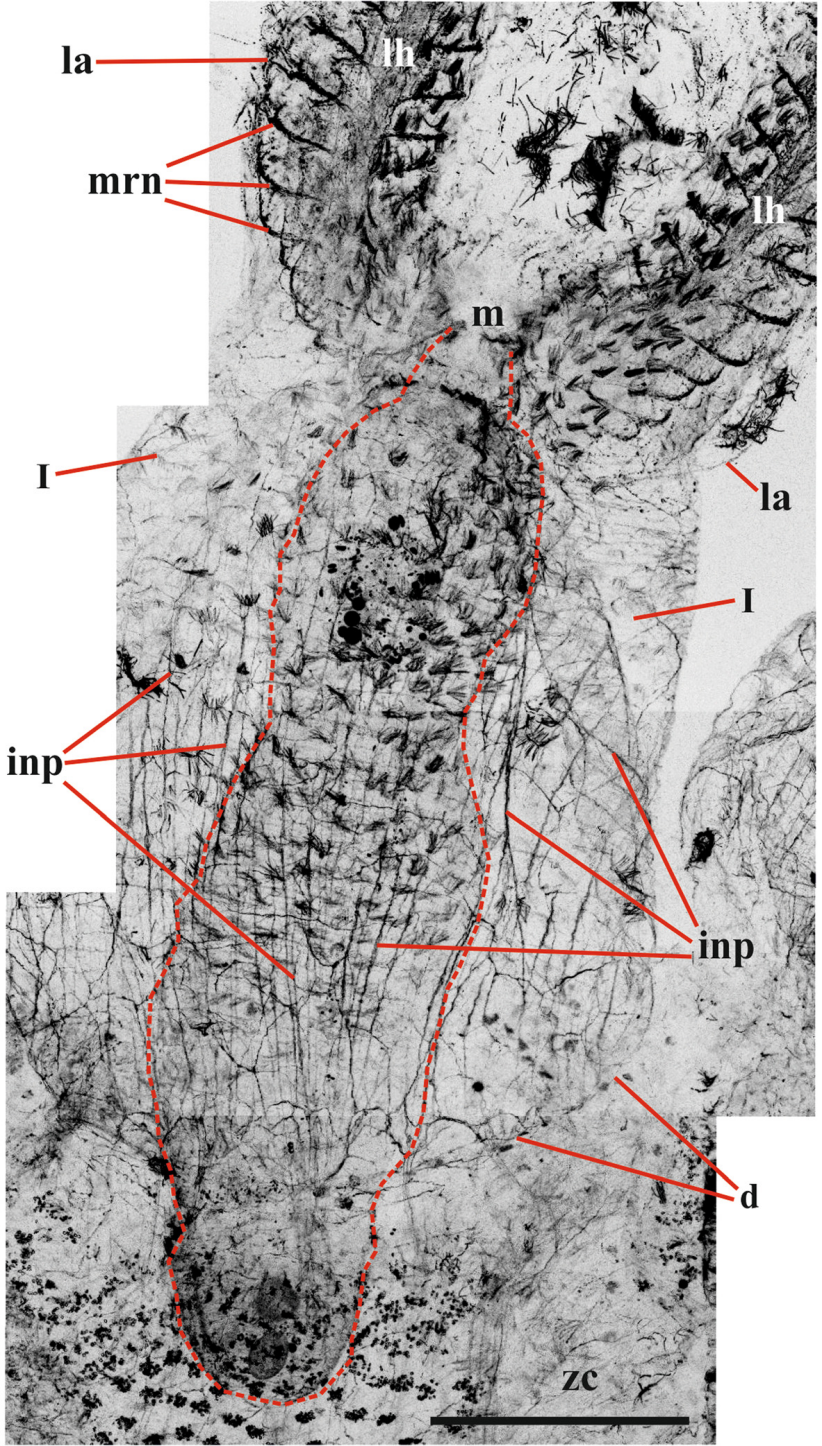
Innervation of lophophore and introvert in *Cristatella mucedo* (viewed from frontal side; pharynx with its ciliature outlined by dashed red line) (staining with antibodies against acetylated tubulin). Abbreviations: d, duplicature; i, introvert; inp, nerve plexus of introvert; la, lophophore arm; lh, lophophore nerve tract (horn); m, mouth; mrn, main radial nerve; zc, zooidal coelom. Scale bar: 200 μm

**Fig. 11:**
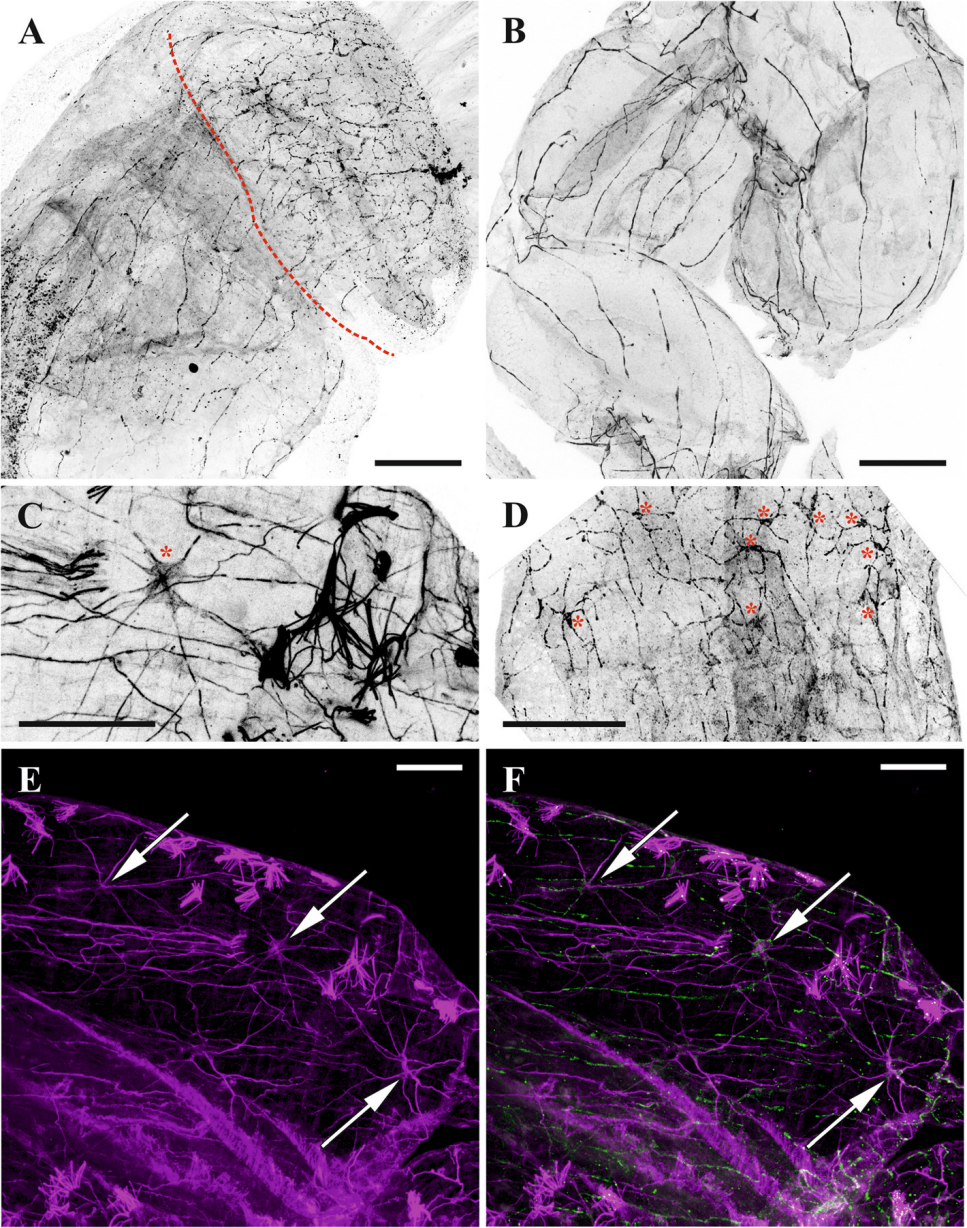
Innervation of introvert and cystid wall in *Plumatella repens* visualized after staining with antibodies against FMRFamide (**a**, **b**, **d**), acetylated tubulin (**c**, **e**) and FMRFamide and acetylated tubulin together (**f**). **a**, nerve plexus of the body wall (partially protruded introvert (above) separated from the cystid by dashed red line); **b**, nerve plexus of cystid wall; **c**, **d**, multipolar neurons in the introvert wall (marked by red asterisks); **e**, **f**, innervation of introvert wall (multipolar neurons shown by arrows). Note that FMRFamide elements often correspond to those revealed by tubulin staining. Scale bars: **a**, **b**, **d**, 100 μm, **c**, **e**, **f**, 50 μm

**Fig. 12:**
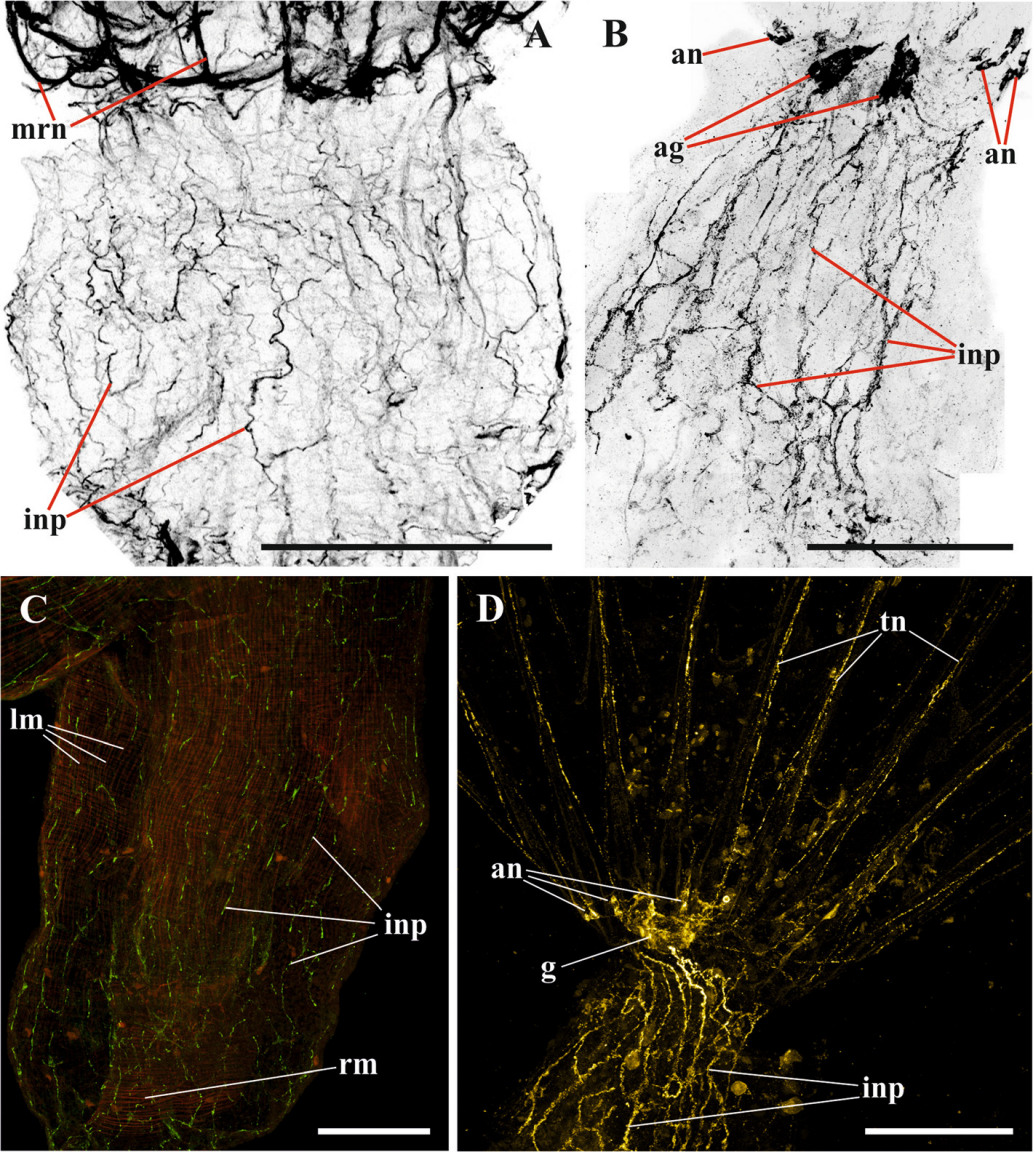
Innervation of introvert and lophophore in *Fredericella sultana* and introvert wall in *Plumatella repens*. **a**, Nervous network of introvert wall in *F. sultana* (lateral view) (staining with antibodies against acetylated tubulin); **b**, cerebral ganglion and nervous network of introvert wall in *F. sultana* (abfrontal view) (staining with antibodies against FMRFamide); **c**, innervation of introvert wall in *P. repens* (staining with antibodies against FMRFamide) (ring muscle layer of the body wall stained by phalloidin is seen); **d**, cerebral ganglion and innervation of introvert wall and lophophore in *F. sultana* (lateral view) (staining with antibodies against FMRFamide). Abbreviations: ag, accumulations of FMRFamide-like immunoreactive neurons in cerebral ganglion near bases of lophophore horns; an, accumulations of neurons at tentacle bases; g, cerebral ganglion; inp, nerve plexus of introvert; lm, longitudinal musculature of introvert wall; mrn, main radial nerve; rm, ring musculature of introvert wall; tn, tentacle nerve. Scale bars: 100 μm

**Fig. 13:**
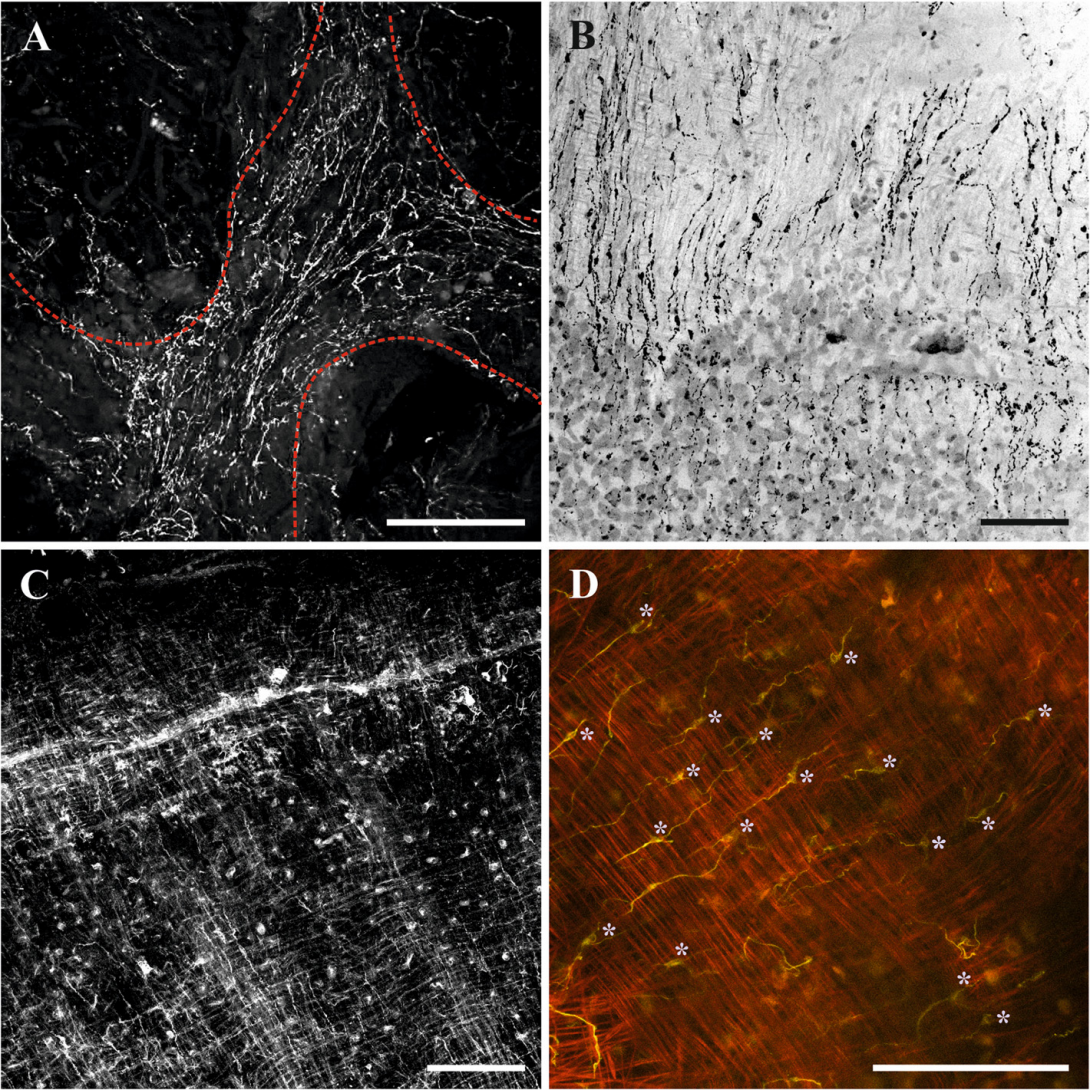
Innervation of cystid wall and sole in *Cristatella mucedo*. **a**, nerve plexus of frontal cystid wall (colony view from above, duplicatures of neighbouring zooids are outlined by dashed red line) (staining with antibodies against FMRFamide); **b**, nerve plexus of sole (bipolar neurons are mostly seen) (staining with antibodies against FMRFamide); **c**, part of sole surrounded by a peripheral rim visible as a thick diagonal line (nerve plexus and two perpendicular muscle layers are clearly seen) (staining with antibodies against acetylated tubulin); **d**, multipolar neurons (marked by asterisks) sandwiched between two perpendicular muscular layers (some of them innervate fibers of both layers) (staining with antibodies against acetylated tubulin and phalloidin). Scale bars: 100 μm

**Fig. 14:**
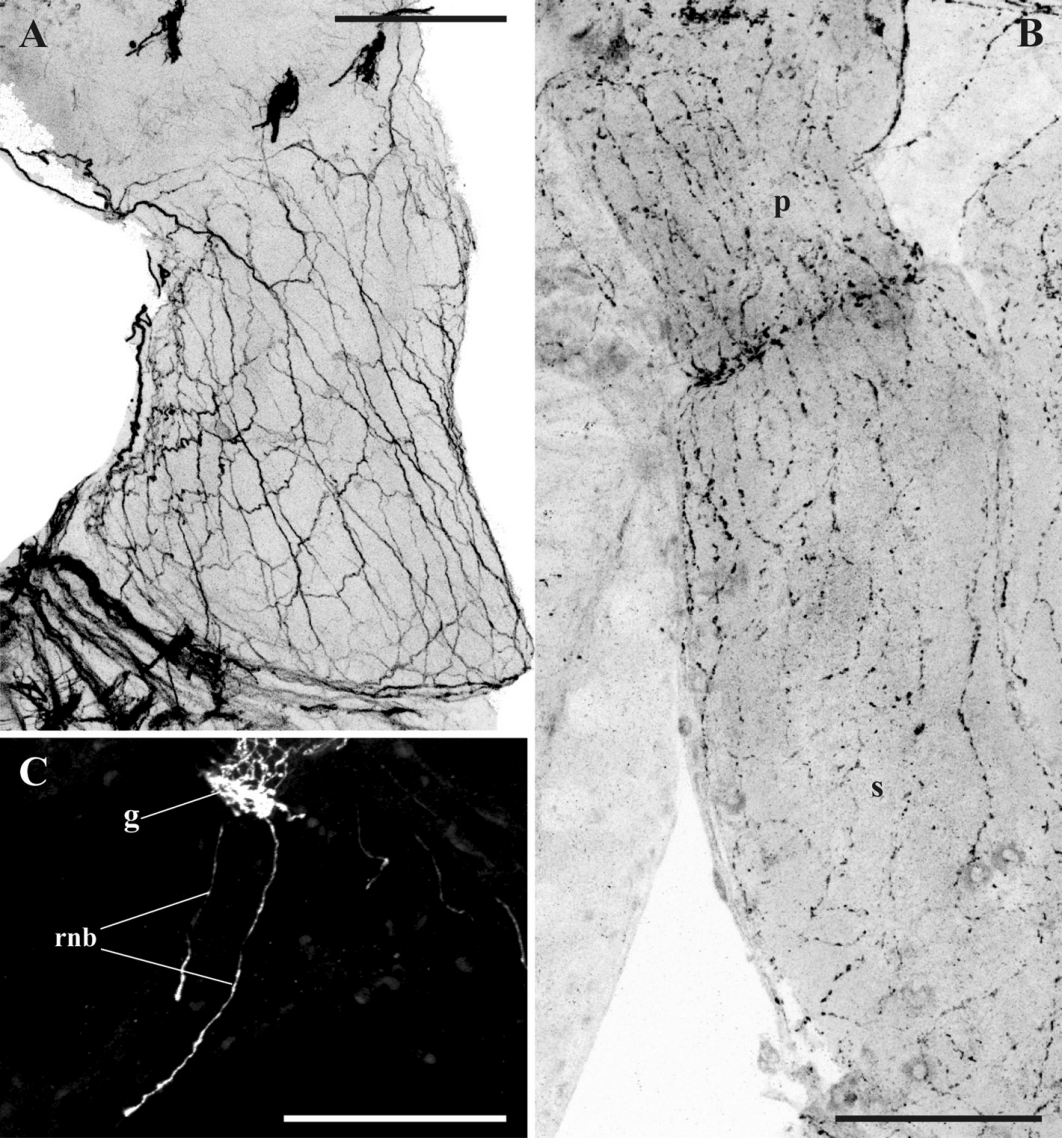
Gut innervation. **a**, nerve plexus of pharynx in *Plumatella repens* (staining with antibodies against acetylated tubulin); **b**, nerve plexus of pharynx (top) and stomach of *Cristatella mucedo* (staining with antibodies against FMRFamide); **c**, two nerve branches emitting from cerebral ganglion and innervating rectum in *C. mucedo* (staining with antibodies against serotonin). Abbreviations: g, cerebral ganglion; p, pharynx; rnb, nerve branches of rectum; s, stomach. Scale bars: 100 μm

**Fig. 15:**
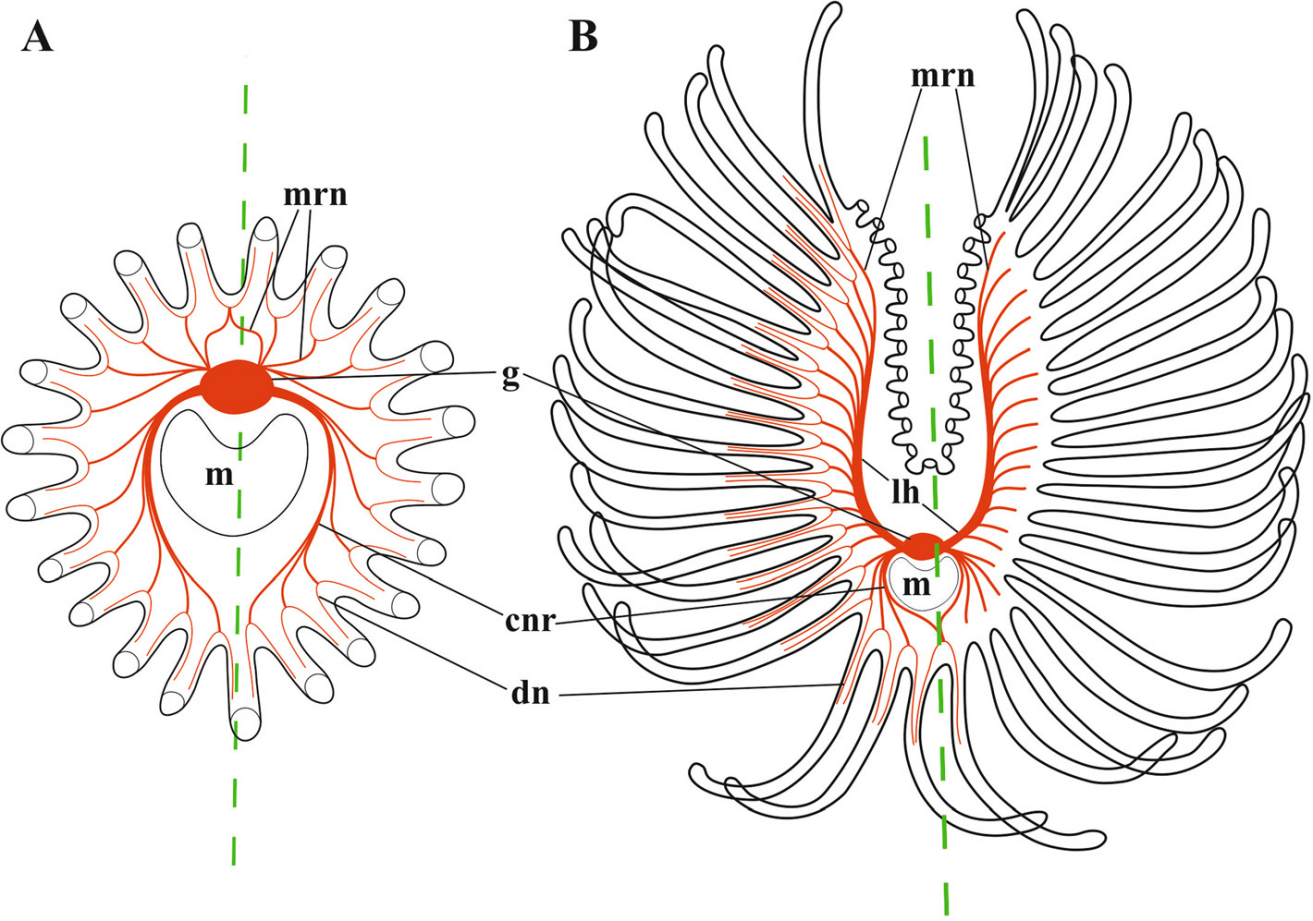
Simplified schemes of lophophore innervation in *Fredericella sultana* (**a**) and *Cristatella-Plumatella* (**b**) based on staining with antibodies against acetylated tubulin. Dashed line corresponds to the fronto-abfrontal (oral-anal) axis. In A only basal parts of tentacles are shown. In B only tentacle bases are shown on the internal sides of lophophore arms. Abbreviations: cnr, circumpharyngeal nerve ring; dn, distal branches of main radial nerve; g, cerebral ganglion; lh, lophophore nerve tract (horn); m, mouth; mrn, main radial nerve

**Fig. 16:**
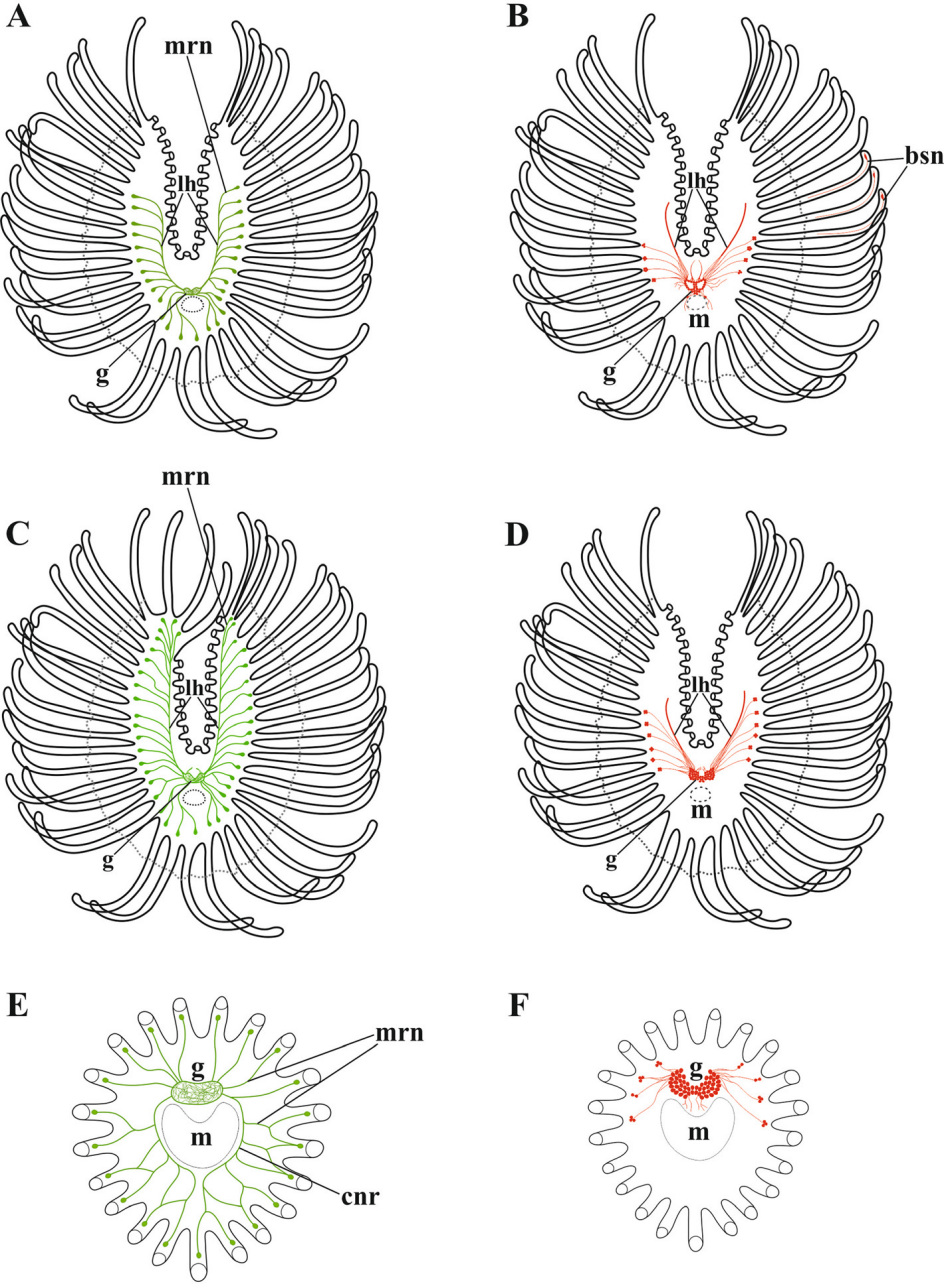
**a**-**f**, Simplified schemes of lophophore innervation in *Cristatella mucedo* (**a**, **b**), *Plumatella repens* (**c**, **d**) and *Fredericella sultana* (**e**, **f**) based on staining with antibodies against serotonin (**a**, **c**, **e**) and FMRFamide (**b**, **d**, **f**) (in (**a**–**d**) only tentacle bases are shown on the internal sides of lophophore arms, intertentacular membrane shown by dashed line; in (**e**, **f**), only tentacle bases are shown). Only perikaryon and central neurite of bipolar neurons at/below tentacle bases are shown. Abbreviations: bsn, bipolar sensory neurons; cnr, circumpharyngeal nerve ring; g, cerebral ganglion; lh, lophophore nerve tract (horn); m, mouth; mrn, main radial nerve

Staining with antibodies against α-tubulin showed that apically the ganglion extends to form two pairs of the large nervous tracts in this species (Figs. 1, 15b, see also Fig. 6c). Two of them that are directed frontally, encircle the pharynx and form the circumpharyngeal nervous ring, whereas the two others are directed abfrontally and form so-called lophophoral “horns” (Figs. 1, 15b, *cnr*, *lh*). These tracts run subepidermally along the mid-line of each lophophore arm and above its coelomic cavity, giving off branches on each side. Finally, three pairs of the nerve tracts arise from the cerebral ganglion basally (proximally). Two larger pairs go laterally through the septum between meso- and metacoel and then ramify densely innervating the introvert and the cystid wall (Fig. 1, *bwn*). The third pair, which is smaller and thinner, goes towards the descending part of the gut, forming its nerve plexus (Fig. 1, pn; for additional details of the digestive tract innervation see also below).

**Fig. 17:**
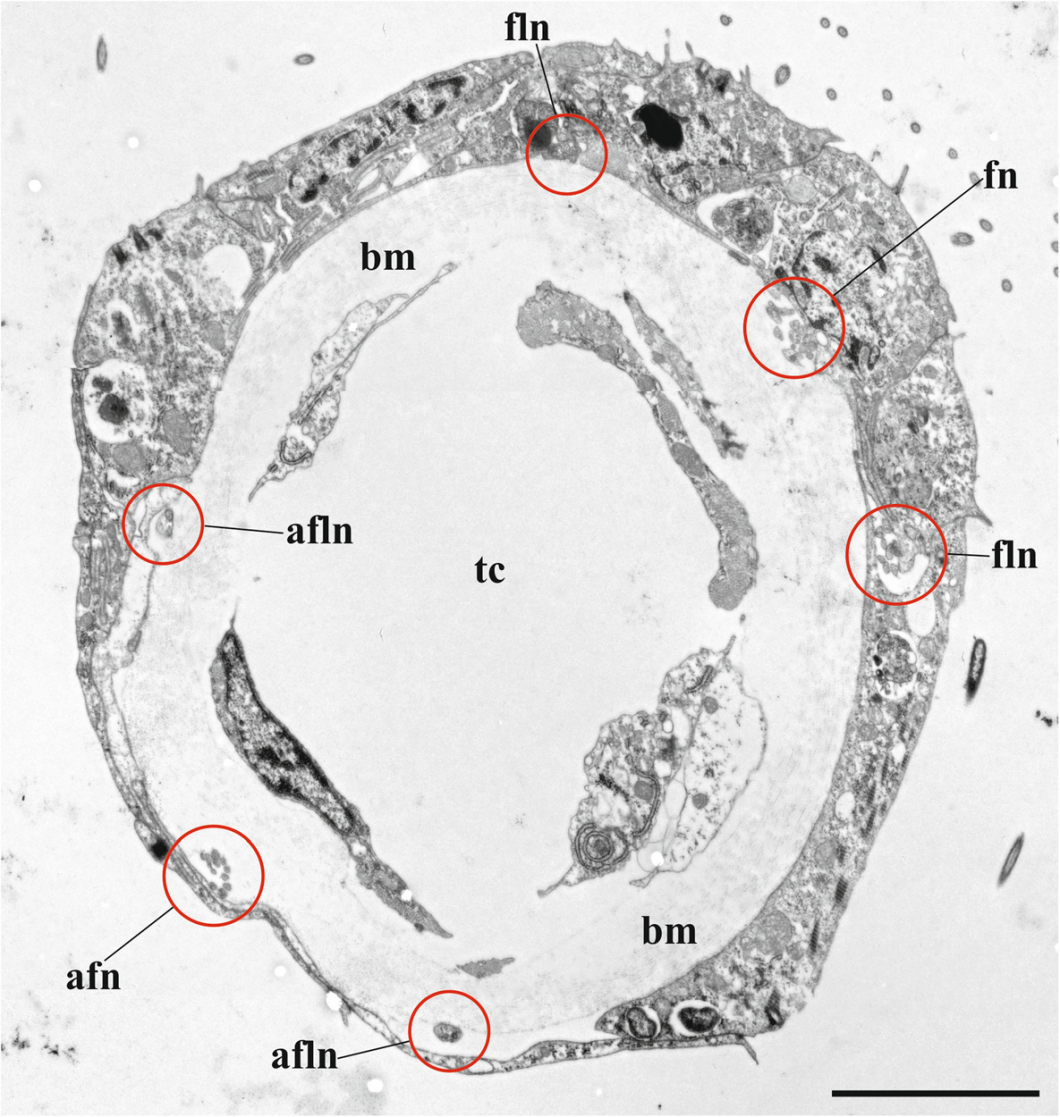
Transverse section of tentacle of *Cristatella mucedo* made across its upper half (TEM). Sections of tentacle nerves are encircled by red. Abbreviations: afln, abfrontolateral nerve; afn, abfrontal nerve; bm, basal membrane; fln, frontolateral nerve, fn, frontal nerve; tc, tentacle coelom. Scale bar: 5 μm

Circumpharyngeal nervous ring and both lophophoral nerve tracts (horns) emit main radial nerves ascending to the intervals between the bases of tentacles – circumoral as well as those placed on both sides of the lophophore arms (Figs. 1, 2, 3, 4, 5d, 10, 15b, *mrn*). Several main radial nerves originate directly from the ganglion, further going towards the intervals between lateral tentacles. Each main radial nerve initially originates (from the ganglion, circumpharyngeal ring or horns) in a horizontal plane further curving up vertically towards tentacles. It ramifies twice on its distal end, sending two pairs of distal branches to two adjacent tentacles (Figs. 1, 2, 3, 4, 5d, 15b, *dn*). One nerve in each such pair is situated closer to the frontal side of the tentacle (facing the ‘inner zone’ of the lophophore arm), further ascending inside it as a tentacle frontolateral nerve (Figs. 4, 17, *fln*). The other nerve of the pair goes to the abfrontal side of the tentacle. It ramifies in the lower third of the tentacle and ascends along the mid-line of its abfrontal side, forming, together with the branches of the neighbouring main radial nerve, the tentacle abfrontal nerve (Figs. 4, 17, *afn*, see also Fig. 6c). The latter complex nerve also includes some strands emitted by the additional radial nerve (see below). Finally, in the middle of the tentacle length the abfrontal nerve branches off two abfrontolateral nerves (Fig. 17, *afln*).

In addition to the distal branching, the main radial nerves emit several fine strands on each lateral side (Figs. 1, 2, 3, 4, *ln*). These lateral branches ascend first to the tentacle base further continuing medially along the entire length of the frontal side of the tentacle, participating in the formation of the complex frontal nerve (Figs. 1, 2, 3, 4, 17, *fn*) that consists of the ‘left’ and the ‘right’ lateral strands of the two neighbouring main radial nerves. The thickness of the lateral strands emitting from the main radial nerves varies: the thick strands can reach up to 3.0 μm in diameter whereas the thin ones are about 1.0 μm. The number of the thick branches is rather stable, reaching 4-5 (on each side of the radial nerve) in *Cristatella mucedo*, 3–4 in *Plumatella repens* and 1–3 in *Fredericella sultana* (Fig. 3). The number of the thin strands is more variable (generally, between 1 and 5) in different species as well as in different lophophores of the same colony. The neighbouring lateral strands are situated rather close to each other in *F. sultana*. The distance between them is a bit larger in *P. repens* and larger still in *C. mucedo*.

Two more nerves arise from the lower third of each main radial nerve. The upper one is an additional radial nerve (Figs. 2d, 4, *arn*) that dichotomously ramifies on the distal end, sending its branches to the abfrontal nerves of two adjacent tentacles and their intertentacular membrane. The second (lower) nerve – basal radial nerve – goes basally and, depending on the part of the lophophore, innervates either the introvert wall or the basal part of the lophophore arm (Figs. 2c, d, 4, *brn*).

In the ganglion of *C. mucedo* serotonin-like immunoreactive elements form a bilaterally symmetrical neuropil consisting of two lobes and a commissure (Figs. 5a, b, *n*, *co*, 16a, *g*). The cell bodies of the bipolar serotonin-like immunoreactive neurons are situated in the lophophore base below the circumoral tentacles (or intervals between them) and below the external tentacle row of the proximal 2/3 of the lophophore arms (Figs. 5a, b, d, *bn*, 16a). Their central neuritеs, as a part of the additional radial nerves, run among the main radial nerves and continue as a part of the circumpharyngeal ring and lophophore horns to the cerebral ganglion. In the lophophore arms these neurites go from the perikarya to the additional radial nerves independently of each other. In the tentacles surrounding the mouth the neurites of the adjacent neurons (including those of the lateral tentacles) merge together within the additional radial nerve before joining the main radial nerve (Figs. 5a, b, d, 16a). Each serotonin-like immunoreactive neuron has a short peripheral projection reaching the tentacle base.

There are two pairs of thin serotonin-like immunoreactive nerves emitting from the cerebral ganglion. One pair is emitted from the apical part of the neuropil and innervates rectum (Fig. 14c,*rnb*). The other pair goes from the basal part of the ganglion to the zooid’s anal side.

In *C. mucedo* 30–40 cell bodies of unipolar FMRFamide-like immunoreactive neurons are located in the peripheral part of the cerebral ganglion. They look like two connected half-rings forming a mask-shaped structure (Figs. 6a, *g*, 16b). A dense accumulation of the cell bodies was seen in the central ‘bar’ of the ‘mask’ as well as in the areas where the circumpharyngeal ring and lophophore horns are emitted from the ganglion. Neurites of these cells go to the neuropil as well as to the horns and circumpharyngeal nerve ring. Small accumulations of FMRFamide-like immunoreactive neurons were also found in the basal part of 4–5 lateral tentacles close to the cerebral ganglion. They usually consist of 3–5 cells (Fig. 6a, b, *an*), and their central neurites go with the main radial nerves to the edges of the mask-shaped structure of the cerebral ganglion or join the lophophoral nerve tracts (Fig. 16b). The peripheral neurite of these cells ascend to the tentacle. One bipolar nerve cell is also situated in the tip of every tentacle of the external row in this species (Figs. 5c, 16b, *bsn*). Its peripheral projection ‘pierces’ the tentacle epithelium whereas the central neurite is included into the frontal tentacle nerve and goes, via the main radial nerve, to the cerebral ganglion. Several FMRFamide-like immunoreactive nerves are emitted abfrontally from the middle part of the cerebral ganglion. They go around the rectum and almost fuse on its back side (Fig. 6b, *nr*). Several branches also originate from the ganglion basally on its frontal (ventral in old literature) side. In particular, one such nerve pair goes down to participate in the innervation of the digestive tract. Positionally it corresponds to the nerve pair stained with antibodies against α-tubulin (Fig. 1, *pn*).

Catecholamine part of the cerebral ganglion is represented by a thick bundle of nerve projections positioned apically on the abfrontal (anal) side of the ganglion. This bundle continues as two nerve cords that soon ramify dichotomously, sending two pairs of branches frontally and abfrontally (Fig. 6c,*g*, *cnr*, *lh*). The former pair participates in the circumpharyngeal ring and the latter pair, in the lophophoral tracts. We failed to find cell bodies in either of the branch described above. In contrast, the cell bodies and the neurites of bipolar sensory cells could be easily seen on our preparations being widely distributed along the entire tentacle length, predominantly on the lateral and laterofrontal tentacle sides (Fig. 6c, *bn*). These cells occur intraepithelially, and their peripheral projection reaches the tentacle surface. The central projection of each such cell is included in either frontal or in frontolateral or in abfrontal tentacle nerves (Fig. 6c, *fn*, *fln*, *afn*). Further these neurites go to both pairs of the distal branches and the lateral strands of the main radial nerves, then to the circumpharyngeal ring or lophophore horns, and finally to the cerebral ganglion. About thirty bipolar (presumably also sensory) neurones are found in the epistome (Fig. 6c). They all lie on its oral surface, whereas their projections form a plexus on the epistome anal side. Catecholamine cells are also found close to the circumpharyngeal ring, supposedly surrounding the mouth. Projections of the multipolar catecholamine neurons form a diffuse plexus in the wall of the descending part of the digestive tract.

The lophophore nervous system of *P. repens* is generally very similar to that of *C. mucedo*. The position and the shape of the cerebral ganglion (70.0 × 30.0 μm in diameter, *n* = 5) as well as the number and the location of the main nerve tracts are the same (Figs. 7, 8a‒c, 15b). The differences revealed by α-tubulin staining include the number and the position of the lateral branches of the main radial nerves (Fig. 3). Whereas in *C. mucedo* the number of such strands is 4–5, *P. repens* has 3–4 strands (see above). Moreover, lateral strands in *P. repens* lophophore are closer to each other than in *C. mucedo*.

Similarly to *C. mucedo*, in *P. repens* serotonin-like immunoreactive part of the neuropil is bilaterally symmetrical, with two lobes and a wide commissure on the frontal side of the cerebral ganglion. A narrow second commissure (sometimes, non-complete) in the abfrontal part of the ganglion was seen in some zooids (Fig. 8a, b, *n*, *co*). On the other hand, the number of serotonergic cell bodies in the lophophore is higher in this species than in *C. mucedo*. Perikarya are found up to the very end of lophophore arms and 3–4 of them are also situated at the tentacles of the lophophore arms internal side. These neuron cell bodies in *P. repens* are situated closer to the tentacle bases than in *C. mucedo* where they lie deeper in the lophophore base (compare Fig. 16a and c). Similarly to *C. mucedo* their central neurites in the anal part of the lophophore go inside the additional radial nerve to the main radial nerves, whereas on the oral side they merge first with a neurite of the neighbouring neuron and then go to the additional radial nerve (Figs, 8a, b, *bn*, 16c).

FMRFamide-like immunoreactive elements in the cerebral ganglion of *P. repens* are represented by a dense accumulation of 30–35 unipolar cell bodies in the peripheral zone. The cells are situated basally on the ganglion’s abfrontal side forming bilaterally symmetrical structure of two interconnected lobes (Fig. 8c). Projections of these neurons are included in the lophophore horns and circumpharyngeal ring. Accumulations of 3–4 FMRFamide-like immunoreactive bipolar neurons are seen at the base of 5–6 lateral tentacles closest to the cerebral ganglion in each arm. Their central neurites go with the main radial nerves to the cerebral ganglion either directly or joining the lophophore horns first (Figs. 8c, and 16d). The peripheral neurites are directed towards the tentacles. At the abfrontal side the cerebral ganglion emits several thin strands that go around the anus. In addition several nerves originate from the basal part of the ganglion innervating the digestive tract and body wall (both introvert and cystid) as in *C. mucedo*.

*Fredericella sultana* has a bell-shaped lophophore with no arms and a circular arrangement of tentacles, which is unusual in phylactolaemates, but otherwise its nerve system is generally similar to that in other freshwater bryozoans. Staining with antibodies against acetylated tubulin revealed two local areas on the abfrontal side of the small ganglion (50.0–60.0 × 30.0 μm in diameter, *n* = 5) (Fig. 15a, *g*) from which two groups of main radial nerves are emitted, a few nerves in each group (Figs. 9a, 15a). These are supposedly rudiments of the lophophoral horns. Two medial-most main radial nerves from each horn merge soon on abfrontal side of the lophophore, uniting in one nerve that ramifies distally twice similarly to all the other main radial nerves (Fig. 15a).

In addition to the reduced lophophore horns, the circumpharyngeal nerve ring in *F. sultana* is incomplete. Its two circumpharyngeal nerve tracts end with the main radial nerves whose distal branches innervate the medial-most ‘frontal’ tentacle (Fig. 15a, see also Fig. 9c showing lophophore innervation visualized after staining with antibodies against serotonin). The remaining parts of the lophophore innervation (additional and basal radial nerves and their distal branches) visualized with the help of α-tubulin staining have the same or very similar structure as in the other two species studied. It should be only mentioned that the lateral branches of the main radial nerves are situated, similarly to *P. repens*, very close to each other (Fig. 3b, c). On the other hand, in *F. sultana* these lateral strands fuse almost immediately after their appearance, and so the frontal tentacle nerve begins much lower in the lophophore base than in the other two species (Figs. 3c, 9a).

In contrast with both *C. mucedo* and *P. repens*, the serotonin-like immunoreactive part of the neuropil is not ‘bilaterally symmetrical’ in *F. sultana*. Instead, it is oval or almost round, being situated in the basal part of the cerebral ganglion on the abfrontal side (Figs. 9d, 16e). Serotonergic cell bodies are situated in the lower third of the tentacles, i.e. much higher than in the other two species described (compare Fig. 16a, c and e). On the abfrontal (anal) side of the lophophore their central neurites are included into the additional and then the main radial nerves, following directly to the neuropil. In contrast, on the frontal side the neurites of the adjacent somas merge together before joining the radial nerves (Figs. 9c, *bn*, 16e). Some of the neurites ramify further fusing with the neighbouring ones to form a rather complex system of roots (Fig. 9c, see similar branching pattern in Fig. 9b visualized after staining with antibodies against acetylated tubulin).

The accumulation of 20–30 FMRFamidergic perikarya is located on the basal surface of the cerebral ganglion in *F. sultana* (Fig. 12d, *g*). This accumulation of neurons is bilaterally symmetrical and consists of two connected lobes that are slightly elongated in the sites where the neurites following to the lophophore horns are emitted from the ganglion (Figs. 12b, d, *ag*, 16f). Projections of these neurons also go to the circumpharyngeal nerves. Small accumulations of usually 2–3 FMRFamide-like immunoreactive cell bodies are found at the base of 3–4 lateral tentacles closest to the cerebral ganglion on each side of the lophophore (Figs. 8d, 12d, *an*, 16f). Their central neurites go with the main radial nerves and further within the circumpharyngeal nerve tracts or reduced lophophore horns to the cerebral ganglion. The peripheral neurite is very long, ascending to the tip of the tentacle. At the oral side short FMRFamidergic nerves are emitted from the ganglion further going to the pharynx wall.

### Tentacle sheath and vestibulum

Apart of two pairs of the thin nerves going from the ganglion to rectum and zooid anal side in *Cristatella*, no serotonin-like immunoreactive elements were found in any other parts of the zooid/colony except the lophophore. The nervous system of the tentacle sheath (introvert) and the vestibulum is mostly represented by FMRFamidergic nerve elements (the amount, shape and position of which correspond in many respects to those revealed by α-tubulin staining, Fig. 10).

The nervous system of these two parts is represented by a nerve plexus that originates from the branches of the basal radial nerves and the basal nerves of the cerebral ganglion. In *Cristatella mucedo* the nerve plexus of the introvert is a dense reticulate network that includes a few multipolar neurons. Many of the thicker neurites are oriented along the introvert apicobasal axis (Fig. 10). In contrast, in the wall of the vestibulum the plexus is sparse with fewer neurons, though the apicobasal orientation of the neurites is similar to that in the introvert.

The pattern of innervation of the introvert and the vestibulum in *Plumatella repens* is almost the same as in *C. mucedo* (Figs. 11a, c‒f, 12c, *inp*). In both species the number of neurons in the tentacle sheath is higher than in the vestibulum, and they are most numerous at the border between these parts of the body wall.

In *Fredericella sultana* there were no visible differences between the innervation of the tentacle sheath and that of the vestibulum. The nervous system of both parts is represented by a dense plexus, thicker nerves going parallel to the longitudinal zooidal axis. No multipolar neurons were found in any part of the body wall (Figs. 8d, 12a, b, d, *inp*).

### Cystid

Similarly to the introvert and the vestibulum, the nerve system of the cystid wall is mainly represented by FMRFamidergic elements in all the three species. In *Plumatella repens* and *Fredericella sultana* the cystid wall possesses a sparse nervous plexus with the vast majority of neurites positioned in parallel to the main zooid axis, which is reminiscent of the innervation of the introvert (Fig. 11a, b). No cell bodies were detected in this region.

In *Cristatella mucedo* the nervous system of the cystid wall consists of the nerve elements of the frontal colony wall (the colony surface between neighbouring polypides) and of the sole. The first part is represented by a highly reticulated plexus with no visible perikarya (Fig. 13a). The sole, comprising the basal wall of the colony, has two perpendicular muscle layers with a network of bipolar and multipolar neurons in between (Fig. 13b–d). The nerve elements of this network are stained by antibodies against both α-tubulin and FMRFamide indicating that most of it has FMRFamide-like immunoreactivity. The neuron somata are positioned on the external muscle layer that is perpendicular to the longitudinal colony axis and corresponding to the cystid wall ring musculature. Some of their neurites are oriented along the external muscle layer, whereas the orientation of the others, innervating the internal (longitudinal) muscle layer, is perpendicular. Thus, the same nervous cells often innervate both musculature layers being sandwiched between them (Fig. 13c, d). The external layer is innervated by fewer neurites than the internal one. Some multipolar neurons were also found on the slightly prominent peripheral rim surrounding the sole. No direct nerve connections between the nerve network of the sole and the central nervous system of zooids were found.

### Digestive tract

In all the three species studied the visceral nervous system is represented by a diffuse nervous plexus, as revealed by the staining with both FMRFamide and α-tubulin (Figs. 6b, *npp*, *npr*, 14a, b). The innervation of the descending part begins as a dense ramification of the smallest (third) pair of the basal nerves emitting from the basal part of the cerebral ganglion (Fig. 1, *pn*). Most of the thicker neurites are oriented along the longitudinal axis of the pharynx whereas the thinner ones go perpendicularly or diagonally. No neuron cell bodies were seen in the plexus. A pair of the thin serotonin-like immunoreactive nerves emitting from the apical part of the neuropil runs towards the rectum (Fig. 14c, *rnb*). Several FMRFamide-like immunoreactive nerves were found to originate from the same part of the ganglion and to go around the rectum.

## Discussion

### Lophophore

Our data considerably added to the existing knowledge of the topography and gross morphology of the nervous system in phylactolaemate bryozoans (Figs. 15, 16) (some preliminary results were recently published in [79–81]). Among other details we found that the additional radial nerve sends its distal branches to the abfrontal nerves of two adjacent tentacles and to the intertentacular membrane between them. Although the additional radial nerves were possibly shown by Gerwerzhagen [46] as consisting of long neurites of the “sensory” cells of the intertentacular membrane (Pl. XIII, Fig. 9), no such cells were revealed by our staining for neuromediators (see also below).

Allman [24] wrote that “the tentacular filaments [main radial nerves] are directed towards the intervals between the tentacula” (p. 31). Following him, Hyatt [27] stated that the “Oral [circumpharyngeal] Branches, pass abdominally, each one half way round the oral aperture throwing off filaments to the bases of the tentacles” (p. 42). In fact, his Pl. 15, Fig. 1 shows that these filaments ascend to the “junctions of the bases of the tentacles”. Hyatt admitted that he could not trace these “filaments” further, but suggested (correctly, as it turned out) that “each one probably splits in two [distal] branches [of the main radial nerves] which climb the approximate sides of every pair of tentacles, one branch on either side” (p. 42) [27]. This idea was supported by Saefftigen [31] who wrote that each radial nerve bifurcated distally sending its branches to two neighbouring tentacles. In contrast, Nitsche [26] thought that the “tentacle nerves” branch inside the intertentacular membrane.

Gerwerzhagen [46] presented a much more detailed picture of *Cristatella mucedo*. According to his description, each main radial nerve bifurcates beneath an interval between two neighbouring tentacles, and each branch thus formed continues to the lateral side of the tentacle as either its "distal" (left) or "proximal" (right) nerve (with a supposed motor function). These nerves correspond to the frontolateral nerves in our study. Additionally Gerwerzhagen described and depicted a “very fine nerve” emitting from each distal branch mentioned above (p. 321, textfig. 3). This nerve fuses with the opposite thin nerve forming a “strand on the external side of the tentacle” (p. 323) (corresponding to our abfrontal nerve). The lateral strands of the adjacent main radial nerves form frontal tentacle nerve (called “Medianplexus”). “Meadianplexus” and the nerve of the tentacle “external side” were supposed to have a sensory function. Thus, in *C. mucedo* each tentacle was described as having four nerves.

Our data confirm the above description that the main radial nerve ramifies twice distally. It should be only added that whereas in each pair one nerve (frontolateral) is thicker than the other (adding to abfrontal) in *C. mucedo* and *F. sultana*, both pairs consist of equally thick nerves in *P. repens*. It should be also noted that the distal ramification of the radial nerve occurs subepidermally in the lophophore wall, and not within the intertentacular membrane as Schwaha and Wood wrote [75].

Marcus [52] wrote that in *Lophopus crystallinus* each main radial nerve emits three nerve pairs (including distal bifurcation) that participate in a formation of five tentacle nerves (all sensory). “Medianen inneren Tentakelnerve” (i.e. frontal nerve) originates by the fusion of the lowest lateral strands of two neighbouring main radial nerves (that corresponds well to both Gerwerzhagen’s and our data), whereas the next pair of the lateral strands gives “inner” nerves (corresponding to frontolateral nerves in our study). The distalmost branches form the “outer” nerves that positionally correspond to abfrontolateral nerves.

The origin of the frontal tentacle nerves from the lateral strands of the main radial nerves was recently shown in *F. sultana* [74].

Combining TEM and CLSM methods we managed to find six nerves in each tentacle of *Cristatella mucedo* originating from the branches of two neighbouring main radial nerves: one frontal (formed by the lateral strands of the main radial nerves), two frontolateral (formed by the distal branches of the main radial nerves), one abfrontal (formed by the distal branches of the main and additional radial nerves) and two abfrontolateral (emitted from the abfrontal nerve) (Fig. 17) [80]. The frontal nerve is an aggregation of 10–15 neurites underlying the basal surface of the frontal epithelial cell(s) in a shallow concavity of the basal lamina. In most cases observed the frontal nerve was ‘flat’ in the cross section, with neurites arranged in 1–3 loose “layers” one on top of the other. In contrast, some frontal nerves were “cylindrical”, with their neurites forming a rather compact group.

Two frontolateral nerves are represented by compact groups of 4–17 (usually 8–12) neurites always running in the ‘canals’ formed by the basal parts of frontal and frontolateral epithelial cells. The lower parts of these cells often form flat overlapping appendages that totally close the ‘entrance’ to such a canal in the cross section. The thin projections of the sensory cells were sometimes seen to enter these canals when joining the frontolateral nerve, which indicates their afferent nature.

The abfrontal nerve is an aggregation of 9–19 neurites embedded into the basal lamina. In some instances few of them underlay the basal surface of abfrontal epithelial cell, whereas in others the entire nerve was surrounded by the lamina’s matrix. Similarly to the frontal nerve, it could be flat or cylindrical in the cross section. Abfrontolateral nerves (originating from abfrontal nerve) are subepidermal. Each of them is represented either by a single and rather wide neurite or 2–3 narrow neurites.

The number and position of the tentacle nerves in *Cristatella mucedo* is reminiscent of the situation in *Asajirella gelatinosa*. In this species Mukai et al. [3] described and depicted seven basepithelial frontal nerves. Among them there are the median frontal nerve and the two peripheral nerves (corresponding to the frontolateral ones in our description), which are thicker than the others. Similarly to *Cristatella*, *Asajirella* has an abfrontal (embedded in the basal lamina) and two basepithelial abfrontolateral nerves. Moreover, the thin nerve fibres were sometimes found between the abfrontal and abfrontolateral nerves (also seen in our Fig. 17). Mukai et al. [3] suggested that while the abfrontal nerve is either motor or mixed, all the other tentacle nerves are sensory. Finally, altogether six tentacle nerves (frontal, two frontolateral, abfrontal and two abfrontolateral) have recently been found by Gruhl (unpubl. data) using CLSM in *L. crystallinus*.

The above data show that the number of the tentacle nerves can differ in different species and different regions of the tentacle. The nerve can also change along the tentacles (e.g. become thinner or branch) and the number of its neurites can change too.

Several authors [27, 34, 46, 52] found two more nerve tracts (the so-called “epistomial nerve ring”) arising from the upper part of cerebral ganglion. According to these authors, several main radial nerves (extending towards the intervals between tentacles of the inner row just behind the epistome) originate from this ring. We failed to visualize the entire epistome nerves; only the short bases of two thick nerves (supposedly epistomial tracts) originating near the bases of the lophophore horns can be seen in some images obtained by α-tubulin staining (Fig. 7a, *en*).

In general, the neuroanatomy of the lophophore is similar in the three studied phylactolaemate species. Though *Fredericella sultana* has a bell-shaped tentacle crown, its lophophore nervous system consists of the same elements as that of phylactolaemates with a horseshoe-shaped lophophore. While elongated tracts of the lophophore arms are missing in this species, there are two lateral areas on the abfrontal side of the cerebral ganglion from which two groups of the main radial nerves are emitted. Judging from the lateral position of these areas and the radiating pattern of the main radial nerves arising from them as two bundles, these structures are likely to be the rudiments of the lophophore horns. This is in agreement with the 3D-reconstruction of the *F. sultana* ganglion made by Gruhl and Bartolomaeus [74], which clearly shows two very short “ganglion horns” (Fig. 2b, c). In fact, these nerve tracts were described already by Hyatt [27] and Braem [34]. Their presence implies that the round lophophore shape is a derived condition, which evolved in freshwater bryozoans independently. This conclusion is confirmed by the molecular data. Hirose with co-authors [82], who used mitochondrial 16S and 12S rRNA gene analysis, showed that fredericellid and plumatellid bryozoans are not the basalmost but the most derived (and sister) groups among Phylactolaemata (see also [83–85]). According to their analysis, the horseshoe-shaped lophophore is primitive and the circular lophophore shape arose later in the evolution [82]. Similarly, the analysis of two nuclear ribosomal and five mitochondrial genes by Waeschenbach et al. [86] implied that the almost circular lophophore in this family could be the result of convergent evolution.

We suggest that the reduction of the lophophore arms and, consequently, an almost entire reduction of the lophophore horns in Fredericellidae is explained by the general miniaturization of zooids in this family as compared to all other Phylactolaemata [81]. Diminishing of zooidal diameter could result in the overall simplification of the lophophore shape accompanied by the reduction of the tentacle size and number in *Fredericella*. This is in agreement with suggestion of Mundy et al. [87] who speculated that the horseshoe-shaped tentacle crown of Phylactolaemata is as a result of the lophophore enlargement.

It should be added that the basal parts of some main radial nerves emitting from these areas form a rather complex system of roots. Similar ‘roots’ of the radial nerves originating directly from the cerebral ganglion were described by Gerwerzhagen [46] in *Cristatella mucedo* and mentioned, even earlier, by Saeffigen [31], but we never saw them in this species.

In addition to the reduced lophophoral horns, we also found that the circumpharyngeal nervous ring in *F. sultana* is incomplete. This could be seen by CLSM on preparations made with the use of all the three staining methods. This observation contradicts to Fig. 2b in Gruhl and Bartolomaeus [74], where a complete circumpharyngeal ring is shown (its completeness is, however, questioned in the text of their paper). Interestingly, an incomplete circumpharyngeal ring in *F. sultana* was depicted by Hyatt (Pl. 15. Fig. 1) [27]. The circumpharyngeal ring also seems to be incomplete in the gymnolaemate *Cryptosula pallasiana*, in which two peripharyngeal tracts do not unite frontally [88]. In the majority of marine bryozoans, however, it seems to be complete [3, 47, 50, 51, 66, 75].

Starting from Gerwerzhagen [46], it was observed that gymnolaemate “oesophagealer Nervenring” (“perioral nerve tracts” of Gordon [88] and “peripharyngeal nerve ring” of Ryland [89]), corresponding to circumpharyngeal ring of Phylactolaemata, consists of the upper and lower ring-like parts. Whereas they were depicted as single nerves in the ctenostome *Amathia verticillata* (as *Z. pellucidum*) by Gerwerzhagen [Textfig. 3 in [47]], Graupner [51] pictured them as the upper wide and lower narrow rings in the cheilostome *Membranipora membranacea* (Textfig. 18). In contrast, the same author as well as Bronstein [53] depicted the single nerve ring in the ctenostomes *Flustrellidra hispida*, *Alcyonidium* sp. and the cheilostome *Bugula calathus*, whereas in the ctenostome *Farella repens* Marcus [50] described the upper “lophophoral” and lower “pharyngeal” nerve rings. The latter picture also corresponds to the data of Lutaud on the cheilostome *Electra pilosa,* whose nervous system was described as having the upper and the lower fascicles of the “peripharyngeal ganglionic belt” [56, 66].

Implementation of the CLSM resulted in the similar situation. Whereas in the ctenostome *Hislopia malayensis* Schwaha and Wood [75] described the “circum-oral nerve ring” consisting of two “circum-oral nerve trunks” connected by a thin bridge at the frontal side, in another ctenstome *Paludicella articulata* Weber et al. [78] found that the “circum-oral nerve ring” is added by the “circum-pharyngeal nerve plexus”. They both originate from the cerebral ganglion. The circum-oral ring surrounds the mouth closing on the side opposite to the ganglion by only a few nerve strands. The circumpharyngeal plexus surrounds the pharynx below the circum-oral ring, and is open on the frontal side. The described diversity may be explained either by interspecific variability or the use of different microscopic techniques (or both), and more research should be done to ascertain this situation.

It is generally accepted that gymnolaemates have four subepidermal tentacle nerves: one frontal, two laterofrontal and one abfrontal (frontal and abfrontal are presumably motor or mixed, whereas laterofrontal are sensory) (reviewed in [3, 66, 78]). According to Lutaud who studied the cheilostome *Electra pilosa* [56, 66] there are short nerves (called “intertentacular” or “axillary forks”) that originate from the upper fascicle of the “peripharyngeal ganglionic belt” (peripharyngeal nerve ring) further dichotomously ramifying to form two “oral sensory” (laterofrontal) nerves of two neighbour tentacles. Frontal and abfrontal tentacle nerves (“motor” and “dorsal sensory” nerves of Lutaud [56, 66]) originate directly from the peripharyngeal ring (its lower fascicle) or from the ganglion.

Graupner [51] presented a similar scheme for the ctenostome *Flustrellidra hispida* (as *Flustrella*) and the cheilostome *Membranipora membranacea*, in which the laterofrontal “sensory” nerves are formed from the intertentacular “forks”, and “motor” tentacle nerves emanate from the peripharyngeal ring. The same branching pattern was described in the lophophore of the ctenostome *Farella repens* by Bronstein [53]. The precise position of the tentacle nerves should be restudied using confocal microscopy and TEM in these species, however, because the two “motor” nerves were described as having lateral subperitoneal position, or, at least, going inside the coelom of the tentacle (also in the ctenostome *Alcyonidium* sp.) [51, 53]. A similar pair of subperitoneal nerves was described by Gordon [88] in *Cryptosula pallasiana,* who also found subepidermal frontal, two laterofrontal and one abfrontal tentacle nerves (six altogether)*.* In contrast, Smith [90] found only subiepidermal nerves in *F. hispida* using TEM, publishing a scheme, which is very similar to the drawings of *Electra pilosa* by Lutaud [56, 66] and those of the cyclostome *Crisia eburnea* by Nielsen and Riisgård [91], who found only four tentacle nerves. It should be mentioned that Smith and Lutaud described neurites passing through the basement membrane towards the muscles in the tentacle cavity.

Recent studies on two other ctenostomes – *Hislopia malayensis* and *Paludicella articulata* - published by Schwaha with co-authors [75, 78] showed the presence of four subepidermal tentacular nerves. The intertentacular nerves (“intertentacular” or “axillary forks” of Lutaud [56, 66]), emitted from the circum-oral nerve ring, bifurcate twice distally. Similarly to the main radial nerves and their distal branches in Phylactolaemata, each “fork” forms four nerves, two of which becoming laterofrontal nerves of the neighbour tentacles whereas two others, fusing with corresponding branches of the neighbour forks, form the abfrontal nerve in two neighbour tentacles. The frontal nerve (“medio-frontal tentacle nerve” in [75, 78] originates directly from the circum-oral nerve ring. This picture clearly differs from what was described in the ctenostomes *F. hispida* and *F. repens* and their careful reinvestigation with modern methods is required.

A scheme totally different from all the above variants is given for *C. pallasiana* where all the four subepidermal tentacle nerves were described as originating from peripharyngeal nerve ring as a bundle, with one nerve separating at the base of the tentacle and going to the abfrontal side of the neighbour tentacle. The subperitoneal nerves arise directly from the nerve ring under the tentacle (see Gordon in Mukai et al. [3]). Thus, the number, position and branching pattern of the tentacle nerves is still under discussion in Gymnolaemata [3]. CLSM and TEM studies involving a wide range of marine bryozoans are needed to clarify this situation. Despite of all uncertainties, a comparison between the tentacle innrevation in Phylacto- and Gymnolaemata shows one major difference. In the former, all the tentacle nerves originate from the intertentacular (radial) nerves, whereas there are one or several tentacle nerves emanating directly from the peripharyngeal nerve ring in the gymnolaemates. Considering Phylactolaemata as a basal bryozoan clade [85, 86], Schwaha with co-authors [75, 78] suggested that these morphological differences characterize a trend towards direct innervation of the tentacles from the peripharyngeal ring in Gymnolaemata. More ctenostome bryozoans should be studied and restudied, however, before making any wider conclusions.

### Cerebral ganglion

Similarly to many other invertebrates [92], phylactolaemate bryozoans possess a cerebral ganglion consisting of the peripheral zone of the nerve cells’ somata and the ‘central’ neuropile of neurites. A unique bryozoan character is a lumen in the neuropil. Earlier scholars, who examined histological sections of different phylactolaemate species, invariably described and depicted the lumen as being rather wide and prominent [29, 30, 34, 35, 46, 47, 51, 52, 93]. On the other hand, Gruhl and Bartolomaeus [74] showed in a TEM study that the lumen in *Fredericella* was slit-like, suggesting that the wide lumen reported by early workers is an artifact of fixation. While it is possible that the tissue did sometimes shrink as a result of fixation, our immunohistochemical images resemble the depictions of the early anatomists who often worked with living animals. For example, the fact that the ganglionic lumen is relatively wide is also supported by the first observations of Allman [23] who managed to recognize this lumen in living specimens and compared it with a “ventricle in its interior” (p. 476).

The lumen of the cerebral ganglion is displaced distally to the frontal side of the ganglion while most of the neuropil and its neuroepithelial ‘cover’ form the abfrontal (anal) and the basal parts of the ganglion. The ganglionic lumen is surrounded by the neuroepithelial cells too [74]. It should be noted that the position of the nerve cell nuclei in the ganglion (peripheral as well as internal, around the lumen) was described as soon as the histological methods were first applied to Phylactolaemata [29, 46, 52, 93].

CLSM made it possible for us to recognize the differences and similarities in the distribution of serotonin-like and FMRFamide-like immunoreactive elements in the ganglion of the three phylactolaemates studied [79]. Serotonergic elements are found in its abfrontal part, predominantly in the neuropil, being represented exclusively by the nerve projections (see also [77]). In *Cristatella mucedo* and *Plumatella repens* these neurites form two bilaterally symmetrical zones interconnected by the central commissure. Both the circumpharyngeal and the lophophoral nerve tracts originate from these symmetrical zones. In contrast, no ‘bilobate’ pattern was revealed in the neuropil of *Fredericella sultana* by serotonin staining, suggesting that the specific distribution pattern found in the former two species is associated with the horseshoe shape of their lophophores. The presence of the commissure could be associated with coordinated activities of the lophophore arms recorded in these species [11, 14, 16]. It is remarkable that an opposite idea that the ganglia of the ‘dual’ “nerve-mass” (p. 80) control independent movements of the lophophore arms was suggested by Hyatt [27].

FMRFamidergic elements are the nerve cells’ somata that are localized in the abfrontal part of the ganglion in its peripheral zone and inside the neuropile. In all the three species studied the neuronal cell bodies are aggregated in the bilaterally symmetrical structure, which is mask-shaped with a central “bar” in *Cristatella* and bilobate in *Plumatella* and *Fredericella*. Such a pattern is presumably associated with a bilaterally symmetrical shape of the lophophore even in the latter case when it is secondarily lost.

In agreement with our observations are two “susoesophagiens” ganglia connected by a commissure that were described and depicted in the earliest papers dealing with the phylactolaemate structure [18–20]. Though Allman [24] and Nitsche [26] described a single ganglion, Hyatt [27] wrote “that there are two ganglia united by a commissure in all the Hypocrepia [phylactolaemates with the horseshoe-shaped lophophore] can hardly be doubtful” (p. 46). An analysis of Hyatt’s text shows that he considered “two swollen lateral ends” of the “nerve-mass” as “two ganglionic centres”. Despite the ganglion’s oval shape, the same ‘dual’ structure was suggested by Hyatt [27] for *Fredericella*. Explaining this contradiction, he stressed, however, that “the size of the commissure … seems to be immaterial … and … of the same thickness as the ganglia themselves” (p. 46). In contrast, in the 3-D reconstruction of the ganglion in *Plumatela imarginata* and *Fredericella sultana* by Gruhl and Bartolomaeus [74] it looks like a tripartite organ consisting of the central and two lateral “masses”.

In Gymnolaemata the cerebral ganglion has the same position on the abfrontal side of the pharynx but its structure is different (for reviews see [3, 66]). In *Electra pilosa* it consists of an eccentric (proximal) neuropil and three cell regions around it: the central one, the distal one from which the peripharyngeal ring begins and the proximal one responsible for the innervation of the body wall and digestive tract [55, 66]. The cerebral ganglion of Cyclostomata consists of just a few cells but its regionalization is presumably the same [61]. Recently a small central lumen surrounded by a neuroepithelium has been found in the ganglion of the ctenostome *Paludicella articulata*. If this ctenostome has, similarly to Phylactolaemata, retained this lumen, it could be an ancestral state in Bryozoa [78].

It should also be noted here that the formation of the ganglion has been recently studied in both Phylacto- and Gymnolaemata by Schwaha with co-authors [75, 94]. Following Davenport [32] and Braem [34, 35] it was proven that the cerebral ganglion is a result of invagination of the abfrontal wall of the foregut in the polypide bud.

Two recent papers based on the CLSM data showed that serotonergic elements were present only in the lophophore and the ganglion of the ancestrula of the cheilostome *Triphyllozoon mucronatum* as well as in the lophophore base of the ctenostome *Hislopia malayensis*. In *T. mucronatum* FMRFamidergic elements were seen only in the base of the lophophore. In both species an elongated cerebral ganglion has a neuropil of serotonergic neurites [73]. In *H. malayensis* there are three serotonergic perikarya at the base of each pair of the tentacles on the oral side of the lophophore; they participate in the peripharyngeal nerve ring. There is also a serotonergic perikaryon between each pair of the remaining tentacles, and its central neurite runs either to the ganglion or to the peripharyngeal nerve ring. Short peripheral neurites extend from each such neuron to two neighbouring tentacles [76].

### Distribution of neuromediators in the lophophore and functions of the tentacle nerves

Gruhl [70] gave some details of the nerve system of the polypide in the larva of *Fredericella sultana.* According to his description, a serotonergic neuropil of the cerebral ganglion is situated near the pharynx. It is worth noting that no serotonin-like immunoreactive perikarya have been found in this area. The nerves originating from the neuropil run towards the tentacles further bifurcating into single neurites. Each neurite terminates in a serotonergic perikaryon at the tentacle base. No FMRFamidergic elements were found in the polypide of the larva.

Schwaha and Wanninger [77] corroborated the data of Gruhl [70] briefly describing the serotonin immunoreactivity in the adult *F. sultana* and three *Plumatella* species including *P. fungosa*. The data of these authors on the ganglion and serotonin elements of the lophophore correspond to our results, although a limited number of images and a very short description do not allow a detailed comparison.

Images obtained by different immunohistochemical staining in our study made it possible to ascertain the distribution of neuromediators in the lophophore and to suggest the function of some neurons and nerves. For instance, as shown by α-tubulin staining, different distal branches of the additional radial nerve participate in the formation of the abfrontal tentacle nerve and go to the intertentacular membrane where they showed a presence of serotonin. Our study did not confirm the presence of sensory cells in the intertentacular membrane of *C. mucedo* reported by Saefftigen [31], Gerwerzhagen [46] and Marcus [52].

The position of the serotonin-like immunoreactive elements in the lophophore of Phylactolaemata suggests that they are mainly afferent. The serotonergic bipolar neurons found in the lophophore underneath the tentacle bases (in *Cristatella* and *Plumatella*) and in the lower third of the tentacles (*Fredericella*) have a short peripheral neurite ending in the tentacle epithelium and the central one running either to the ganglion or the circumpharyngeal ring or lophophore tracts seemingly without any connection to muscles.

In contrast, the distribution of the FMRFamide-like immunoreactivity in the introvert and cystid wall as well as the digestive tract suggests that these nerve elements are mostly efferent. FMRFamide-like immunoreactive neurites are often oriented along the muscle bundles including the nerve plexus in the sole of *C. mucedo* found in between two muscle layers. On the other hand, a bipolar FMRFamidenergic neuron in the tip of every tentacle in this species is obviously sensory. Its peripheral projection ‘pierces’ the tentacle epithelium whereas the central neurite runs together with the frontal tentacle nerve to the cerebral ganglion. A similar terminal cell with the long neurite and presumably with a sensory function has been described by Graupner [51] in the tentacle tip of the gymnolaemate bryozoan *Membranipora membranacea*.

In all the three species studied small groups of FMRFamide-like immunoreactive neurons occur in the basal part of 3–5 lateral tentacles. Their central neurites go with the main radial nerves to the cerebral ganglion or join the lophophore tracts. Although similar cell groups were described by Gerwerzhagen [46] as sensory in *C. mucedo*, their function is yet unclear.

Mukai et al. [3] suggested that the abfrontal nerve may be motor or mixed, whereas all other tentacle nerves are sensory in Phylactolaemata. This suggestion is supported by the fact that the frontal tentacle nerve includes catecholamine and FMRFergide neurites of intraepithelial cells located at the lateral/frontolateral sides and on the tip of the tentacle correspondingly. The position of the catecholamine bipolar neurons whose central projection participates in the frontal, abfrontal or frontolateral nerves corresponds to the laterofrontal cells with a sensory cilium described in *Plumatella* sp. by Gilmour [95] and in *F. sultana, C. mucedo* and *Lophopus crystallinus* by Riisgård et al. [96, 97]. Gerwerzhagen [46] and Marcus [52] obviously described the same “sensory cells of the tentacle” [46] (p. 322). Presumably, it is the thin projections of these cells that were seen to join the frontolateral nerves in our TEM images. The abfrontal nerve is embedded to the basal lamina, which can indicate that some of its neurites are motor, as suggested by Mukai et al. [3] for *Asajirella*. On the other hand, Phylactolaemata possess median abfrontal sensory cells [96, 97], and the corresponding tentacle nerve, as suggested above, can have a mixed function. Abfrontolateral nerves might also be connected with the median sensory cells but this supposition is entirely speculative at the moment.

In gymnolaemate bryozoans both the frontal and abfrontal tentacle nerves are supposedly motor or mixed (since their fibres sometimes pass through the basal lamina into the epitheliomuscular cells of the tentacle) whereas laterofrontal nerves are sensory (summarized in [3]) (see also above).

### Tentacle sheath and vestibulum

In all the three species studied the introvert and vestibulum are innervated by the reticulate nerve plexus. It originates from two short nerve pairs (“dorsal” and “ventral” motor nerves of Gerwerzhagen [46]), which go laterally from the basal part of the cerebral ganglion towards the oral side of the lophophore and then densely ramify in the introvert and the cystid wall. Some basal radial nerves of the lophophore innervate the introvert too.

In *Cristatella mucedo* and *Plumatella repens* multipolar neurons occur in this plexus in both the tentacle sheath and the vestibulum. No such neurons are found in these parts of the body wall of *Fredericella sultana*. Many thicker neurites are oriented along the longitudinal axis of the introvert, thus corresponding to the internal (longitudinal) muscle layer. In contrast, perpendicularly oriented neurites could innervate the outer layer of the circular musculature, which was recently described by Schwaha and Wanninger [77] by CSLM. We suggest that some of these neurites could be connected with peripheral mechanoreceptors, whereas others have efferent function. A review of the body wall innervation in Gymnolaemata can be found in [3, 66, 78].

### Cystid

Differences in the innervation of the cystid wall in the three species studied may be explained by the shape of the cystid, whose distal part is tubular in *Plumatella* and *Fredericella*. In these species most of the neurites of the nerve plexus are oriented in parallel to the longitudinal zooidal axis. In contrast, the cystid wall of *Cristatella mucedo* has no such tubular part and is characterized by a highly reticulated plexus with no predominant orientation of neurites. We suggest that similar ‘diffuse’ pattern should be also present in the brood chambers – invaginations of the cystid wall [98, 99] – of all phylactolaemates regardless of the cystid shape.

Verworn [28] was one of the first to describe creeping in small young *C. mucedo* colonies. Since that time several researchers observed it in young colonies of *Lophopus* and *Pectinatella*. Experiments were made and possible locomotion mechanisms, e.g. lophophore excursions and rigidity of epithelial cells, were suggested (reviewed by Marcus [48, 49]). Based on the data of Gerwerzhagen [46], who described the continuous nerve plexus in the body wall of *Cristatella*, Hyman [5] suggested that “a ganglionated plexus that has some anastomoses with the plexus of the tentacle sheath through the muscle layer and that is spread throughout the colony, … [explains] the coordinated creeping peculiar to this genus” (p. 451). Moving colonies were reported to have a negative phototaxis [49] though photoreceptors in them are unknown. Indeed, an extensive network of the multipolar neurons found in between two perpendicular muscle layers of the sole should have a motor function. There are multipolar neurons on the peripheral rim of the sole, but whether they have a sensory function is unknown. We suggest that these cells can be involved in recognition of the laminar water currents and the locomotion of *Cristatella* is a response to a surrounding hydroregime rather than to the light.

## Conclusions

Our research is the first extensive CLSM study of comparative neuromorphology of phylactolaemate bryozoans. It resulted in a comprehensive picture showing the structure and distribution of the main nervous system elements in a zooid as well the distribution of neuromediators. The general architecture of the nerve system is similar in the three species under study but a number of differences were also found. We speculate that the secondary simplification of the lophophore in *Fredericella sultana* was accompanied in the course of evolution by corresponding changes in its nerve system, mainly the reduction of the lophophore arms. These changes may be, in general, associated with the reduction in size.

The use of various methods in combination shed light on the distribution and function of the tentacle nerves. Whereas the frontal and frontolateral nerves are supposedly sensory, the abfrontal one seemingly has a mixed nature. The function of abfrontolateral nerves is currently unclear.

Despite the basic similarity, the nervous system of both the ganglion and the lophophore in Phylactolaemata is noticeably more complex than that in both Gymno- and Stenolaemata. The neuronal network has a denser and a more complex branching pattern and consists of more neurons. This can be, in part, explained by the horseshoe shape of the lophophore and a generally larger polypide size (more 1 mm) in the freshwater bryozoans. In its turn, a larger zooidal size can be associated with a generally calmer hydroregime: life in predominantly still water could require the evolution of the larger food-capturing apparatus in Phylactolaemata.

Interestingly, the structure of the nerve system in *Fredericella* is still rather complex and includes more elements in comparison with the representatives of the other two bryozoan classes despite the round shape of the lophophore. Observations on a wide range of the behavioural reactions are necessary to compare them with the diverse polypide behaviour described in marine Bryozoa by Winston [7, 8] and Shunatova and Ostrovsky [12]. Such observations might show if a more complex neuromorphology correlates with a more diverse/complex behaviour. According to preliminary data of Antipenko [11], *F. sultana* behaves similarly to the marine species.

The nerve plexus sandwiched between two muscular layers of the sole in *Crystatella mucedo* is supposedly represented by the peripheral motor neurons. How their activity is initiated and co-ordinated is still unknown since no photoreceptors are found in phylactolaemates. We suggest that creeping of *Cristatella* can be associated with a ‘search’ for the optimal hydroregime rather than with the light avoidance.

The extensive data obtained in our research only highlight the fact that neuromorphology of Phylactolaemata, as well as that of Gymno- and Stenolaemata, is understudied. Comparative research across a wide range of species from different families is necessary.

## Materials and methods

Colonies of *Cristatella mucedo*, *Plumatella repens* and *Fredericella sultana* were collected in 2009–2012 in the pools of the Petrodvortsovyi District of St. Petersburg. The colonies were anesthetized by the drop by drop addition of 10 % MgCl_2_ solution and then fixed in a 4 % paraformaldehyde solution buffered with 0.1 M phosphate buffered saline (PBS). After fixation the specimens were washed 3 × 20′ in PBS with 0.1 % Triton X-100 (PBT) and proceeded to immunohistochemical staining immediately or stored in PBS with 0.1 % NaN3 at +4 °С. For immunohistochemical staining the specimens were blocked overnight in PBT with 1 % bovine serum albumin. Subsequently monoclonal antibodies against acetylated α-tubulin (Sigma, T6793) combined with polyclonal antibodies against FMRFamide (Immunostar, 20,091) or serotonin (Immunostar, 20,080) were applied for 24 h. The primary antibodies were diluted 1:500 – 1:2000 in PBT. After the incubation specimens were washed 3 × 20′ in PBT, incubated overnight with secondary fluorochrome-conjugated antibodies (goat anti-rabbit Alexa Fluor ® 488 (Invitrogen, A-11,008) and donkey anti-mouse Alexa Fluor ® 647 (Invitrogen, A-31,571) diluted 1:500 – 1:1000 in PBT), and then washed 3 × 20′ in PBT. All the incubations were performed at +4 °С [76].

We also stained the muscular elements using TRITC-phalloidin (diluted 1:100) buffered by PBS (pH = 7.4). After 1–2 h in phalloidin colonies were washed 3 × 10′ in PBS. Nuclei were additionally stained by HOECHST 33,258 (H1398, Invitrogen) buffered by PBS during 15 min. For visualization of catecholamines polypides obtained from anesthetized colonies were stained by 9.2 % solution of glyoxylic acid prepared on 10 % sucrose during 30 min (+4 °С) and dried.

After staining the single polypides and the colony pieces were embedded in 97 % TDE, 80 % glycerol with PBS or vaseline oil, correspondingly, and studied under confocal laser scanning microscopes (CLSM): Leica TSC SPE (University of Bonn, Germany) and TCS SP5 (Saint Petersburg State University, and Zoological Institute, Russian Academy of Sciences). The images obtained were processed using the ImageJ software.

For transmission electron microscopy colonies of *C. mucedo* were fixed in 1.25 % glutaraldehyde (on 0.1M cacodylate buffer, pH 7.4) during 1 h [74], then washed 3 × 20′ in 0.1M cacodylate buffer (pH 7.4) and postfixed with 1–2 % solution of osmium tetroxide (OsO_4_) followed by three rinses, each 20 min, in distilled water. After washing, polypides were dehydrated in an acetone series and subsequently embedded in plastic (Araldite + EPON) being further dissected. Ultrathin sections 70 nm were placed on formvar-coated single-slot copper grids and stained with uranyl acetate and lead citrate using an automatic TEM stainer RMC Products QG-3100. Sections were analyzed using TEM microscopes ZEISS Libra 120 (University of Bonn, Germany) and Jeol JEM-2100 (Saint-Petersburg State University).

## Additional file

Additional file 1:
**History of the research on the bryozoan nervous system in XIX-XX centuries.** (DOC 70 kb)
