# Supplementary material for: Comparative morphology of the nervous system in three phylactolaemate bryozoans
Source: Front Zool. 2015 Oct 12;12:28. doi: 10.1186/s12983-015-0112-2 (PMC4603689; doi:10.1186/s12983-015-0112-2)
Supplement: Additional file 1: — History of the research on the bryozoan nervous system in XIX-XX centuries. (DOC 70 kb) [file 12983_2015_112_MOESM1_ESM.doc]

Additional file

**History of the research on the bryozoan nervous system in XIX-XX centuries**

From the first published description of a living bryozoan by Trembley [1] to the last quarter of the 19^th^ century, bryozoans were studied as total preparations (alive of fixed) without staining. The earliest observations of the nerve elements in Phylactolaemata was published by Dumortier [2] who described and depicted two supraoesophagal ganglia connected by a commissure and called them “brain” in living zooids of *Lophopus crystallinus* (p. 432, Pl. 6, fig. 5). Both ganglia were described as oval and having a reniform shape in a lateral view (Pl. 6, fig. 2). Dumortier wrote that they are expanded into the cavity, but we can only speculate whether he meant the lophophore nerve tracts running to the coeloms of the arms of the horseshoe lophophore.

In contrast, Van Beneden [3,4] wrote about a single ganglion in his papers dealing with *Fredericella* and *Plumatella* (as *Alcyonella*). He also mentioned a complete [circumpharyngeal] ring. Confusingly, two ganglia connected by a wide commissure are described repeatedly in the joint papers of Dumortier and Van Beneden [5,6] on *Plumatella* (as *Alcyonella*) and *Lophopus*. It is clearly visible on their pictures (Pl. IV, figs. 3, 5, 7), however, that the ganglion is single, being described as “deux ganglions” because of its dumbbell-like shape [5]. These authors also mentioned and depicted a complete circumpharyngeal ring and described nerves going from the ganglion to the epistome, retractor muscles and oesophagus in *Plumatella*. In *Fredericella* the nervous system was described as a round semi-transparent organ that “can be considered, because of its position, as a nerve ring” (p. 30). In their Pl. III, fig. 3, however, only on oval “ganglion nerveux” was shown [5].

Allman described a ganglion lying on the “rectal aspect… of the oesophagus, … with a cavity or ventricle in its interior” in *Plumatella* spp. (as *Alcyonella*) (p. 476) [7]. Further he wrote: “from each side [of the ganglion] may be seen passing off a rather thick chord” that “takes a course backwards and immediately enters the tubular arms of the lophophore” further running “along the roof of the [coelomic] tube, giving off at regular intervals a filament to each tentacle upon the outer margin of the arm”. Further Allman stated that “when [the lophophore nerve tract] arrives at the extremity of the arm it turns on itself, and in its retrograde course gives off similar filaments to the tentacula placed upon the inner margin. It finally terminates by uniting with its fellow…”. In this paper Allman mentioned that some nerves from the upper edge of the ganglion go towards “the mouth and its valvular appendage [epistome]”, saying that he was not able to see the “perfect [nerve] collar around this tube [pharynx]”.

The same doubts one can see in his monograph published in 1856, although according to Allman’s depiction of the lophophore of *Lophopus crystallinus* (Pl. II, fig. 24) the cerebral ganglion issues [supposedly on each lateral side] a nerve that is connected with a continuous nerve running around the pharynx and further along the lophophore arm sending filaments to the intervals *between* the tentacles [8].

The ganglion and several lophophoral nerves were also detected by Hancock [9] in *Fredericella* and *Plumatella*. Nitsche [10] clearly stated that the nerve system of *Plumatella fungosa* (as *Alcyonella*) consists of a ganglion, a circumpharyngeal ring, lophophore tracts (that he called horns) and their nerves ascending between tentacles to the intertentacular membrane. The depicted optical section of the lophophore arm (Tab. 3, fig. 29) shows that the horn occurs on the roof of the lophophore arm coelom. Kafka [11] used the data of Nitsche in his review.

The most detailed description of the phylactolaemate nervous system at that time was published by Hyatt [12] who, similarly to his predecessors, worked on living animals. Hyatt’s account is correct in most details, and it is truly amazing that such a precise description could be achieved without making sections and staining the nerve tissue. While it is not always clear what he meant, this author described “anterior and posterior sets” of the paired nerve branches (larger “nerve-trunks” and “their smaller ramifications”, p. 41) emitting from the “nerve mass” [ganglion] to the epistome, lophophore, introvert and gut. In *Fredericella* sp. (as *F. regina*), for instance, the position and branching pattern of the “Oral” [circumpharyngeal] and “Branchial Branches” [rudiments of the lophophore horns] were explained and schematically depicted together with the explanation of differences in the innervation of the dorsal and ventral sides of the lophophore. These details of the nervous structure were compared with those in *Plumatella* sp. (as P. *diffusa*) and *Pectinatella magnifica*. Viewed from the above, the ganglion was depicted as oval in *Fredericella* and dumbbell-like in *Plumatella*. Viewed from the lateral side, it was described as “thin, upright and spindle-like” and “kidney-shape[d], laying horizontally” correspondingly in these phylactolaemates. Incidentally, Hyatt was the first to mention interzooidal variation in the branching pattern of the nerves originating from the ganglion.

Histological methods invented in the third part of the 19^th^ century greatly benefited anatomical studies. Verworn [13], Kraepelin [14,15], Saeffigen [16] and Braem [17,18] described the nerve system of several phylactolaemate species. In particular, the latter three authors observed that in the cerebral ganglion the nerve cells form a peripheral layer surrounding the thin nerve threads [neuropil], and that the ganglion lumen expands to the bases of the lophophore horns (up to one third of the arm length in *Plumatella*). Saeffigen also stated that each tentacle is innervated by the distal branches of two neighbouring nerves (that he called “Radial”) originating from the lophophore horns. Supporting observations of Hyatt [12], Braem [17] was the first to describe and picture the general schemes of the lophophore nerve system in both horseshoe and circular phylactolaemate lophophores, showing that, though circular, it still has the short “Lophophorarmnerven” [horns] in *Fredericella* (p. 66, Tab. V, fig. 66). Davenport [19] and Braem [17,18] carefully studied the formation of the cerebral ganglion showing that this process occurs via invagination of the incipient foregut wall. Some data on the nerve system of *Asajirella gelatinosa* (as *Pectinatella*) were obtained by Oka [20].

The most careful and important study of the phylactolaemate nervous system was undertaken by Gerwerzhagen [21,22] who used histological sections and vital staining by methylen blue. This author described all the major nerve elements of the lophophore, the digestive tract and the body wall in *Cristatella* and made suggestions concerning their function. He was followed by Marcus [23-25] who was the first to reveal the nerve elements in a phylactolaemate larva. Marcus [25] also studied the neuromorphology of an adult bryozoan (*Lophopus crystallinus*) and found some details (for instance, the branching pattern of the main radial nerves, the number of the tentacle nerves, etc.) to be different from the descriptions of Gerwerzhagen [21]. Graupner [26] studied anatomy, histology and development of the ganglion and lophophore horns in *L. crystallinus* adding many important details to the early descriptions. Finally, Brien [27] included his original data on the nerve system of *Plumatella fungosa* in his review on Bryozoa.

Studies on the nerve system of marine gymnolaemate Bryozoa began when Van Beneden [28,29] described the cerebral ganglion in the ctenostomes *Farella repens* (as *Laguncula*) and *Alcyonidium* sp. This author could not recognize the circumpharyngeal nerve ring but had no doubts about its existence. Several other authors described the details of the neuroanatomy (predominantly, position and shape of the ganglion) as a by-product of their taxonomical or anatomical research [14,30-34]. Calvet described the ganglion and the peripharyngeal nerves on histological sections [35].

The study of Gerwerzhagen [22] revealed more details in the structure of the gymnolaemate nervous system. This author used vital staining by methylen blue as he did with Phylactolaemata. Gerwerzhagen was the first to show the ‘double’ nature of the peripharyngeal ring and discovered the tentacle nerves and the nerve tracts running from the ganglion to the digestive tract and the introvert wall. Marcus [23,36], Graupner [26], and Bronstein [37] greatly added to the existing data, and Hiller [38] described the visceral and so-called “colonial” [in fact, that of the cystid wall] nervous system in Gymnolaemata.

A milestone in neuromorphological research on marine bryozoans are the works of Lutaud [39-49] who used various staining methods as well as the transmission electron microscopy (TEM). Many of the Lutaud’s findings were included in her reviews [50,51], which updated previous compilations [27,52,53]. Two more reviews on the bryozoan nervous system were written by Bullock and Horridge [54] and Ryland [55]. The latter paper included TEM data on the tentacle nerves of some gymno- and stenolaemates obtained by Smith [56], Gordon [57] and Nielsen and Riisgård [58]. Finally, a detailed updated review on the comparative neuromorphology of Bryozoa, with addition of the original ultrastructural data on phylactolaemate *Asajirella gelatinosa*, was published by Mukai et al. [59].

Summarizing the above written, it should be stressed that despite rather considerable number of published papers, the general picture of the bryozoan neuromorphology is still incomplete and fragmentary. In part, it is because of the poor taxonomic coverage. Also, the data of the old authors should be thoroughly checked and their descriptions revised using modern techniques. Altogether, much more work should be done before our knowledge of bryozoan nervous system will be adequate.

**References:**

1. Trembley A : *Memoires Pour Servir a l'Histoire d'Un Genre de Polypes d'Eau Douce, a Bras en Forme de Cornes*. Paris: Jean and Herman Verbeek; 1744.
2. Dumortier BC: **Recherches sur l’anatomie et la physiologie des polypiers composés d’eau douce**. *Bull Acad R Sci, Bruxelles* 1835, **2**:421-454.
3. van Beneden PJ: **Quelques observations sur les Polypes d'eau douce**. *Bull Acad R Sci, Bruxelles* 1839, **6**:276-279
4. van Beneden PJ: **Recherches sur les Bryozoaires ﬂuviatiles de Belgique**. *Nouv Mem Acad R Belg* 1848, **21**:1-33.
5. Dumortier BC, Van Beneden PJ: **Histoire naturelle des polypes composés d'eau douce: II Partie. Descriptions**. *Bull Acad R Sci, Bruxelles* 1848, **16** (complement):1-96.
6. Dumortier BC, van Beneden PJ: **Histoire naturelle des polypiers composés d'eau douce des bryozoaires fluviatiles**. *Bull Acad R Sci, Bruxelles* 1850, **16** (extrait):1-130.
7. Allman GJ: **On the natural history of the genus *Alcyonella***. *Proc R Irish Acad* 1847, **4**:470-478.
8. Allman GA: *Monograph of the Fresh-water Polyzoa, Including All the Known Species, Both British and Foreign*. London: Ray Society; 1856.
9. Hancock A: **On the anatomy of the freshwater Bryozoa with descriptions of three new species**. *Ann Mag Nat Hist* 1850, **5**:173-202.
10. Nitsche H: *Beiträge zur Anatomie und Entwickelungsgeschichte der phylactolaemen Süsswasserbryozoen, insbesondere von Alcyonella fungosa Pall*. Inaugural Dissertation, Berlin: Friedrich Wilhelms Universität; 1868.
11. Kafka J: **Die Süßwasserbryozoen Böhmens**. Arc Nat Land Böhmen 1887, **6**:1–74.
12. Hyatt A: **Observations on Polyzoa. Suborder Phylactolaemata**. *Proc Essex Inst* 1866-1868, **4-5**:1-103.
13. Verworn M: **Beitrage zur Kenntis der Susswasserbryozoen**. *Z Wiss Zool* 1887, **46**: 99-130.
14. Kraepelin K: **Die Deutschen Süsswasser-Bryozoen. Anatomish-systematischer Teil**. *Abh Naturwiss Ver Hamburg* 1887, **10**:1-168.
15. Kraepelin K: **Die Deutschen Süsswasser-Bryozoen. Entwicklungsgeschichtlicher Teil**. *Abh Naturwiss Ver Hamburg* 1892, **12**:1-67.
16. Saefftigen A: **Das Nervensystem der phylactolaemen Susswasser-Bryozoen**. *Zool Anz* 1888, **11**:96–99.
17. Braem F: **Untersuchungen über die Bryozoen des süssen Wassers**. *Bibl Zool* 1890, **6**:1-134.
18. Braem F: **Die Keimung der Statoblasten von *Pectinatella* und *Cristatella***. *Zoologica (Stuttgart)* 1913, **26**:35-64.
19. Davenport CB: ***Cristatella*: The origin and development of the individual in the colony**. *Bull Mus Comp Zool Harvard Univ* 1890, **20**:101-151.
20. Oka A: **Observations on fresh-water Polyzoa (*Pectinatella gelatinosa*, nov. sp.)**. J Coll Sci Imp Univ Japan 1891, **4**:89-150.
21. Gerwerzhagen A: **Beiträge zur Kenntnis der Bryozoen. 1. Das Nervensystem von *Cristatella mucedo* Cuv**. *Z Wiss Zool* 1913a, **107**:309-345.
22. Gerwerzhagen A: **Untersuchungen an Bryozoen**. *Sitz Heidelb Akad Wiss, Math-nat Kl B* 1913b, **9**:1-16.
23. Marcus E: **Bryozoa**. In *Biologie der Tiere Deutschlands. 14*. Edited by Schulze P. Berlin: Bornträger; 1925, **47**:1-46.
24. Marcus E: **Beobachtungen und Versuche an lebenden Süßwasserbryozoen**. *Zool Jahrb Syst* 1926a, **52**:279-350.
25. Marcus E: **Über *Lophopus crystallinus* (Pall.)**. *Zool Jahrb Abt Anat Ontog Tiere* 1934, **58**:501-606.
26. Graupner H: **Zur Kenntnis der feineren Anatomie der Bryozoen (Nervensystem, Muskulatur, Stützmembran)**. *Z Wiss Zool* 1930, **136**:38-97
27. Brien P: **Classe des Bryozoaires**. In *Traité de Zoologie*. Edited by Grassé P-P. Paris: Masson; 1960:1053-1379.
28. van Beneden PJ: **Recherches sur l´organisation des *Laguncula* et l´histoire naturelle des différents polypes Bryozoaires qui habitent la côte d´Ostende**. *Nouv Mem Acad R Belg* 1844a, **18**:1-29.
29. van Beneden PJ: **Recherches sur l’anatomie, la physiologie et le développement des bryozoaires qui habitent la cote d’Ostende**. *Nouv Mem Acad R Belg* 1844b, **18**:1-44.
30. Nitsche H: **Beiträge zur Kenntnis der Bryozoen. III. Über die Anatomie und Entwicklungsgeschichte von *Flustra membranacea***. *Z Wiss Zool* 1871, **21**:37-91.
31. Ehlers E: ***Hypophorella expansa*. Ein beitrag zur Kenntniss der minirenden Bryozoen**. *Ab Konig Ges Wiss Göttingen Phys Cl* 1876, **21**:3-157.
32. Vigelius WJ: **Die Bryozoen, gesammelt während der dritten und vierten Polarfahrt des “Willem Barents” in den Jahren 1880 und 1881**. *Bijdr Dierkunde* 1884, **11**:1-104.
33. Vigelius WJ: **Contributions à la morphologie des bryozoaires ectoptoctes**. *Tijds Nederl Dierk Ver*, 2 Series 1887, **1**:77-92.
34. Ostroumoff A: **Contribition à l’étude zoologique et morphologique des Bryozoaires du Golfe de Sebastopol**. *Arch Slav Biol* 1886, **2**: 8-25, 184-190, 329-355.
35. Calvet L: **Contribution à l’histoire naturelle des Bryozoaires Ectoproctes marins**. *Trav Inst Zool Univ Montpellier*, Nouvelle Série 1900, **8**:1-488.
36. Marcus E: **Beobachtungen und Versuche an lebeden Meeresbryozoen**. *Zool Jahrb Abt Syst Okol Geogr Tiere* 1926b, **52**:1-102.
37. Bronstein G: **Étude du système nerveux de quelques Bryozaires Gymnolémides**. *Trav Stat Biol Roscoff* 1937, **15**:155-174.
38. Hiller S: **The so-called nervous colonial system in Bryozoa**. *Nature* 1939, **143**:1069-1070.
39. Lutaud G: **Le «plexus» pariétal de Hiller et la coloration du système nerveux par le bleu de méthylène chez quelques Bryozoaires Chilostomes**. *Z Zellf Mikr Anat* 1969, **99**:302-314.
40. Lutaud G: **L’innervation du lophophore chez le Bryozoaire chilostome *Electra pilosa* (L.)**. *Z Zellf Mikr Anat* 1973a, **140**:217-234.
41. Lutaud G: **The great tentacle sheath nerve as the path of an innervation of the frontal wall structures in the cheilostome *Electra pilosa* (Linné)**. In *Living and Fossil Bryozoa*. Edited by Larwood GP. London, New York: Academic Press; 1973b:3l7-326.
42. Lutaud G: **Le plexus pariétal des Cténostomes chez *Bowerbankia gracilis* (Leydi)**. *Cah Biol Mar* 1974, **15**:403-408.
43. Lutaud G: **L’innervation des parois de la loge chez *Flustra papyracea* (Ellis et Solander) (Bryozoaire Chilostome)**. *Cah Biol Mar* 1976, **17**:337-346.
44. Lutaud G: **L'innervation de l'aviculaire pédonculé des Bicellariidae (Bryozaires Chilostomes)**. *Cah Biol Mar* 1977a, **18**:435-448.
45. Lutaud G: **The probability of a plexus in the calcified wall of *Crisidia cornuta* (Linné)**. In *Advances in Bryozoology*. Edited by Larwood GP, Abbot MB. London: Academic Press; 1979a:33-45.
46. Lutaud G: **Étude ultrastructurale du plexus coloniale et recherche de connections nerveuses interzooidales chez le bryozoaire chilostome *Electra pilosa* (Linné)**. *Cah Biol Mar* 1979b, **20**:315-324.
47. Lutaud G: **The innervation of the external wall in the carnosan ctenostome *Alcyonidium polyoum* (Hassall)**. In *Recent and Fossil Bryozoa*. Edited by Larwood GP, Nielsen C. Fredensborg: Olsen and Olsen; 1981:143-150.
48. Lutaud G: **La structure et I’innervation du diaphragme de la gaine tentaculaire chez deux Bryozoaires Malacostéges**. *Zool Scr* 1984, **13**:59-67.
49. Lutaud G : **L’innervation sensorielle du lophophore et de la région orale chez les Bryozoaires Cheilostomes**. *Ann Sci Nat Zool Biol Anim* 1993, **14**:137-146.
50. Lutaud G: **The bryozoan nervous system**. In *Biology of Bryozoans*. Edited by Woolacott RM, Zimmer RL. New York: Academic Press; 1977b:377-410.
51. Lutaud G: **Autozooid morphogenesis in anascan cheilostomates**. In *Treatise on Invertebrate Paleontology, Part G (Revised), Bryozoa*. Edited by Robison RA. Boulder, Colorado: Geological Society of America; Lawrence: University of Kansas; 1983: 208-237.
52. Cori CJ: **Bryozoa. Ordnung der Tentaculata**. In *Handbuch der Zoologie, III*. Edited by Kukenthal W, Krumbach T. Berlin: W de Gruyter; 1941, **2**:263-374, 375-502.
53. Hyman LH: *The Invertebrates: Smaller Coelomate Groups. Volume 5: VIII*. New York: McGraw-Hill; 1959.
54. Bullock TH, Horridge GA: **Lophophorate phyla: Ectoprocta, Brachiopoda, and Phoronida**. In *Structure and Function in the Nervous System of Invertebrates, Volume 1.* Editor by Bullock TH, Horridge GA. San Francisco: WH Freeman; 1965:631– 647.
55. Ryland JS: **Physiology and ecology of marine bryozoans**. In *Advances in Marine Biology*. Edited by Russell FS, Yonge CM. Academic Press: London/New York; 1976, **14**:285–443.
56. Smith LW: **Ultrastructure of the tentacles of *Flustrellidra hispida* (Fabricius)**. In *Living and Fossil Bryozoa*. Edited by Larwood GP. London, New York: Academic Press; 1973:335-342.
57. Gordon DP: **Microarchitecture and function of the lophophore in the bryozoan *Cryptosula pallasiana***. *Mar Biol* 1974, **27**:147-163.
58. Nielsen C, Riisgård HU. **Tentacle structure and filter-feeding in *Crisia eburnea* and other cyclostomatous bryozoans, with a review of upstream-collecting mechanisms.** *Mar Ecol Prog Ser* 1998, **168**:163-186.
59. Mukai H, Terakado K, Reed CG: **Bryozoa**. In *Microscopic Anatomy of Invertebrates, Volume 13*. Edited by Harrison FW. New York: Wiley-Liss; 1997:45-206.
